# Characterizing inhibitors of human AP endonuclease 1

**DOI:** 10.1371/journal.pone.0280526

**Published:** 2023-01-18

**Authors:** Lakshmi S. Pidugu, Hardler W. Servius, Spiridon E. Sevdalis, Mary E. Cook, Kristen M. Varney, Edwin Pozharski, Alexander C. Drohat

**Affiliations:** 1 Department of Biochemistry and Molecular Biology, University of Maryland School of Medicine, Baltimore, Maryland, United States of America; 2 Center for Biomolecular Therapeutics, Institute for Bioscience and Biotechnology Research, Rockville, Maryland, United States of America; Weizmann Institute of Science, ISRAEL

## Abstract

AP endonuclease 1 (APE1) processes DNA lesions including apurinic/apyrimidinic sites and 3´-blocking groups, mediating base excision repair and single strand break repair. Much effort has focused on developing specific inhibitors of APE1, which could have important applications in basic research and potentially lead to clinical anticancer agents. We used structural, biophysical, and biochemical methods to characterize several reported inhibitors, including 7-nitroindole-2-carboxylic acid (CRT0044876), given its small size, reported potency, and widespread use for studying APE1. Intriguingly, NMR chemical shift perturbation (CSP) experiments show that CRT0044876 and three similar indole-2-carboxylic acids bind a pocket distal from the APE1 active site. A crystal structure confirms these findings and defines the pose for 5-nitroindole-2-carboxylic acid. However, dynamic light scattering experiments show the indole compounds form colloidal aggregates that could bind (sequester) APE1, causing nonspecific inhibition. Endonuclease assays show the compounds lack significant APE1 inhibition under conditions (detergent) that disrupt aggregation. Thus, binding of the indole-2-carboxylic acids at the remote pocket does not inhibit APE1 repair activity. Myricetin also forms aggregates and lacks APE1 inhibition under aggregate-disrupting conditions. Two other reported compounds (MLS000552981, MLS000419194) inhibit APE1 *in vitro* with low micromolar IC_50_ and do not appear to aggregate in this concentration range. However, NMR CSP experiments indicate the compounds do not bind specifically to apo- or Mg^2+^-bound APE1, pointing to a non-specific mode of inhibition, possibly DNA binding. Our results highlight methods for rigorous interrogation of putative APE1 inhibitors and should facilitate future efforts to discover compounds that specifically inhibit this important repair enzyme.

## Introduction

Mammalian AP endonuclease 1 (APE1) initiates repair of abasic (apurinic/apyrimidinic) sites and other toxic and mutagenic DNA lesions and performs critical roles in base excision repair (BER) and single strand break repair (SSBR) [1, 2]. APE1 hydrolytically cleaves phosphodiester bonds at abasic sites, which arise through spontaneous rupture of the *N*-glycosyl bond (depurination) or by the activity of DNA glycosylases, which initiate BER [3, 4]. APE1 is the major mammalian enzyme for repair of abasic sites, which impair DNA replication and other processes, causing DNA strand breaks and cross links. In addition to its AP endonuclease activity, the exonuclease activity of APE1 processes BER intermediates generated by bifunctional DNA glycosylases and removes 3´-blocking groups arising at strand breaks [5, 6].

APE1 repairs DNA lesions generated by clinical anticancer agents, countering the effects of ionizing radiation (IR) and drugs including alkylating agents (temozolomide), antimetabolites (5-fluorouracil), and bleomycin [7–10]. APE1 is elevated in cancers and this correlates with increased tumor progression, decreased survival and reduced sensitivity to IR and chemotherapy [8, 11–16]. Suppression of APE1 repair activity, through genetic depletion or small molecule inhibitors, sensitizes tumor cells to anticancer agents [6–10, 17–19]. Other studies suggest APE1 inhibitors could serve as standalone agents for treating BRCA- or PTEN-deficient cancers [20–22], in a synthetic-lethal approach similar to that established for inhibitors of poly(ADP-ribose) polymerase (PARP) [23, 24], which is also a BER enzyme. Inhibitors of APE1 could also provide important tools for dissecting its various functions in cells or animal models. In addition to the DNA repair activities noted above, APE1 participates in active DNA demethylation through the TET-TDG-BER pathway (Ten-eleven translocase, thymine DNA glycosylase) [25]. Because APE1 is essential for cell viability and has multiple functions, suppressing its expression is not an ideal approach for reducing its repair activity. Rather, small molecule inhibitors could offer a selective, rapidly reversible method to suppress APE1 repair activity.

**Fig 1:**
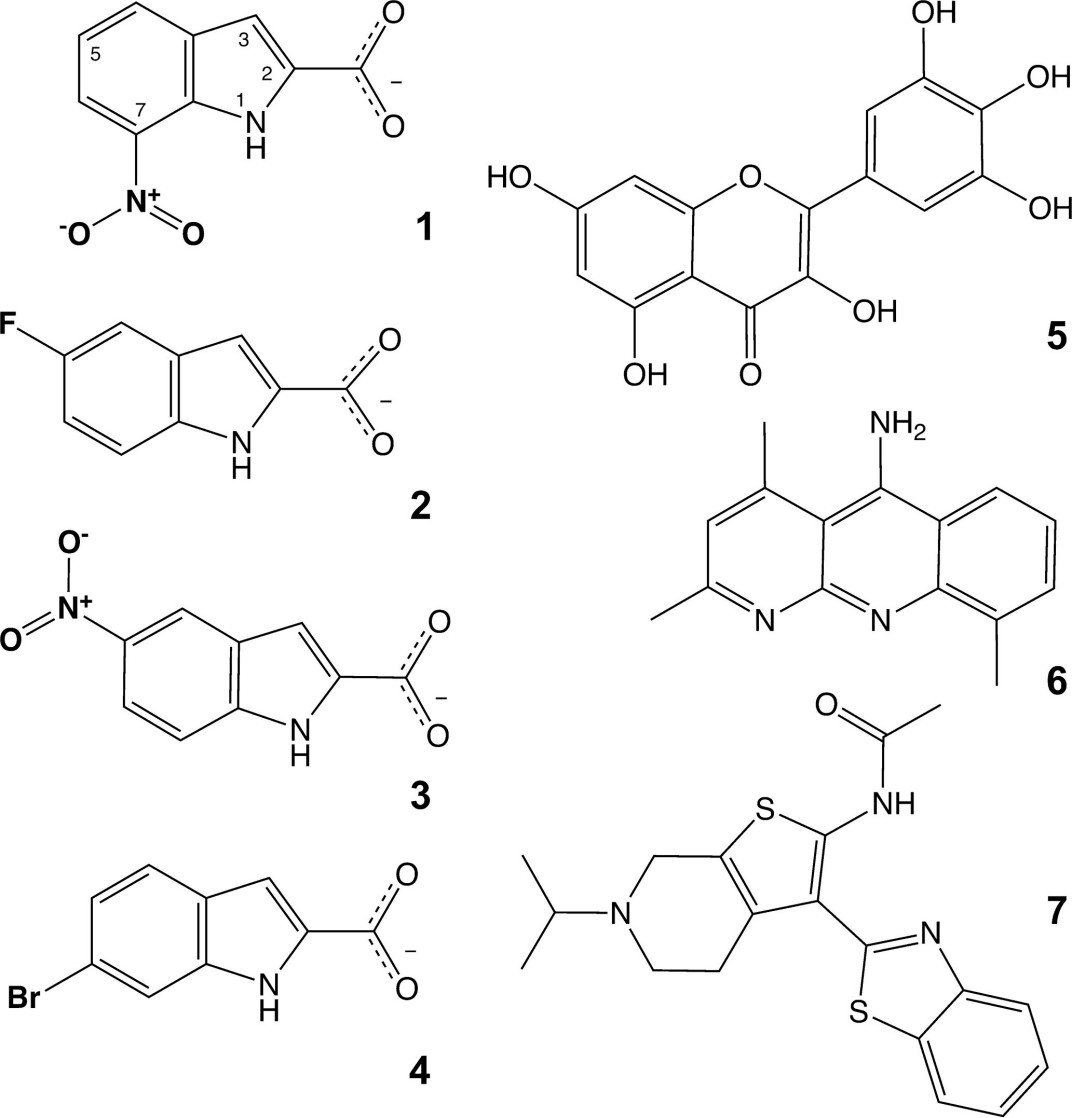
Compounds studied in this work. **1**, 7-nitroindole-2-carboxylic acid or CRT0044876; **2**, 5-fluoroindole-2-carboxylic acid; **3**, 5-nitroindole-2-carboxylic acid; **4**, 6-bromoindole-2-carboxylic acid; **5**, myricetin; **6**, AR03 or MLS000552981 of the NIH MLSMR library; **7,** APE1 Inhibitor III, MLS000419194, or N-(3-(benzo[d]- thiazol-2-yl)-6-isopropyl-4,5,6,7-tetrahydrothieno[2,3-c]- pyridin-2-yl)acetamide. For compounds **1**–**4**, the 2-carboxyl is in the deprotonated form that likely predominates at pH 7.5.

Previous studies identified many compounds that inhibit APE1, *in vitro* and in human cells, as summarized in reviews [26, 27]. Prior studies often included controls and secondary assays to scrutinize hits for artifactual effects, such as interference in a fluorescence-based screen or DNA intercalation, but the studies did not report investigations to ascertain whether the compounds form colloidal aggregates, which could potentially sequester APE1 and cause nonspecific inhibition [28–30]. Compound aggregation is a prominent source of false positives in high throughput screening, which can remain undetected in secondary assays unless experiments are performed to directly investigate aggregation. In this study we used structural, biophysical, and biochemical methods to examine some of the more prominent and widely used APE1 inhibitors, and some related compounds (Fig 1). We sought to determine whether the compounds form colloidal aggregates and to characterize their effect on APE1 activity under conditions that allow or disrupt compound aggregation. We also investigated the APE1 binding site for these compounds using NMR chemical shift perturbation (CSP) experiments, and for one compound, X-ray crystallography. Our findings provide new insight into the efficacy of previously reported APE1 inhibitors and are expected to inform future efforts to develop novel inhibitors of this important BER enzyme.

## Materials and methods

### Materials

Compounds **1**, **4**, **5**, and **7** were obtained from Sigma, **2** and **3** from Alfa Aesar (through Fisher), and **6** from Axon Medchem. BSA (ultrapure, non-acetylated) was obtained from Invitrogen. Human APE1 lacking the 38 N-terminal residues (APE1^ΔN38^), uniformly ^15^N-labeled, was expressed in *E*. *coli* (37°C, 4 h) and purified essentially as described [31, 32]. Purification included Ni-affinity chromatography, thrombin cleavage of the N-terminal poly-His tag, followed by ion-exchange and size exclusion chromatography. The protein was >99% pure by SDS-PAGE and its concentration was determined by absorbance (ε^280^ = 54.4 mM^–1^ cm^–1^). Oligodeoxynucleotides (ODNs) were obtained from IDT, purified by reverse phase HPLC. The duplex DNA used for APE1 endonuclease assays included a target strand, 5´-*TTA CCA GTC CGT CAC C*F*G TAC AGA GCT GAT CC, where *T is fluorescein-dT and *F* is tetrahydrofuran (abasic site analog) and a complementary strand, 5´-GGA TCA GCT CTG TAC GGG TGA CGG ACT GGT A. ODNs were, exchanged into 0.02 M Tris-HCl pH 7.5, 0.04 M NaCl, quantified by absorbance at 260 nm and used to prepare duplex DNA substrates [33].

### Enzyme activity assays

To determine the Michaelis-Menten parameters for APE1 endonuclease activity, kinetics experiments were collected at room temperature (23°C) in a reaction buffer comprised of 50 mM Tris-HCl pH 7.5, 0.1 M NaCl, 0.2 mM DTT, 1 mM EDTA, and 2 mM MgCl_2_ and included 0.1 mg/ml BSA and 0.05% Brij 35 (unless otherwise noted). The truncated form of APE1 (APE1^ΔN38^) was required for NMR studies to avoid strong signals from the disordered N-terminal region that overwhelm resonances of the structured domain and thereby hinder the shift perturbation experiments [32]. APE1^ΔN38^ was used for structural studies because crystallization conditions are optimized for this construct and the disordered residues are not observed in crystal structures. Because the N-terminal region does not contribute to the endonuclease activity of APE1 [34–36], and for consistency in the multidisciplinary studies here, we used APE1^ΔN38^ for activity assays. Reactions were performed under multiple turnover conditions, with an APE1 concentration of 0.01 nM and substrate concentrations of 10 nM and higher. The reactions were initiated by adding concentrated MgCl_2_ to a solution containing all other components. At various time points samples (45 μl) were extracted and quenched with 5 μl of 10x quench solution (1 M NaOH, 0.1 M EDTA). Control reactions demonstrated the absence of activity for samples that contained all components except MgCl_2_. The fraction product for a given sample was determined by UHPLC under denaturing (pH 12) conditions, similar to those we have reported [37]. The alkaline conditions melt the DNA duplex and the resulting ODNs (intact and cleaved) are resolved by anion exchange using a DNAPac PA200 RS column (Thermo). ODN elution was monitored by absorbance (260 nm) and fluorescence (5´-fluorescein-dT). We used fluorescein linked to thymine of a terminal (5´) dT nucleotide because APE1 can excise fluorophores linked by a phosphodiester bond to the ODN terminus [38]. Peak integrals were used to determine the fraction of abasic strand cleaved by APE1, giving fraction product. The initial velocity (*v*_0_) was determined by fitting progress curves (product concentration versus time) to a linear equation using samples from the initial linear region (<10% product). The dependence of *v*_0_ on substrate concentration was fitted to the Michaelis-Menten equation using non-linear regression (Grafit 5) to obtain *k*_cat_ and *K*_M_.

Reactions in the presence of a given compound were performed using the same conditions with a DNA substrate concentration of 80 nM and varying concentrations of the compound. In all cases, the compound was introduced from a 100x stock prepared in DMSO, giving a final DMSO concentration of 1%. Samples containing all components (except MgCl_2_) were incubated for 30 min at room temperature, and activity was initiated by adding MgCl_2_ (2 mM final). Fraction activity (FA) is given by the ratio of initial velocity (*v*_0_) in the presence and absence of compound (FA = *v*_0_^cpd^/*v*_0_^DMSO^, where *v*_0_^cpd^ and *v*_0_^DMSO^ are the initial velocities in the presence of compound or DMSO (1%) control. The dependence of FA on compound concentration ([I]) was fitted to Eq 1,

$$\text{FA}=range\;/\;\left(1+{\left(\left[\text{I}\right]/{\text{IC}}_{50}\right)}^{s}\right)$$ (1)

giving the IC_50_, the range of FA, and the slope factor (*s*).

### Dynamic light scattering

Dynamic light scattering (DLS) experiments were performed to monitor compound aggregation, using a SpectroLight 610 instrument (Xtal Concepts, Hamburg). The instrument features a laser diode (λ 658 nm) which was set to a power of 100 mW and a scattering angle of 150°. For each sample, five replicates of 60 sec duration were collected at 20°C. Instrument settings, including integration time, were held constant for all experiments. Samples (1 ul) were prepared under mineral oil in 96 well SBS plates. Samples included the same buffer used for enzyme activity assays, with or without Brij 35 (and in all cases without BSA). The compounds were introduced from 100x stocks prepared in DMSO, giving a final DMSO concentration of 1%; samples without compound contained 1% DMSO. The DLS studies were in some cases performed using compound stocks that had been stored at –20°C in DMSO. Control experiments performed with fresh 100x stocks in DMSO showed that, upon dilution, the compounds formed aggregates at the same concentrations as samples which had been stored at –20°C, indicating that the freeze-thaw process did not cause the observed aggregation.

### NMR spectroscopy

NMR samples (0.55 mL) contained uniformly ^15^N-labeled APE1^ΔN38^ (0.05 mM to 0.15 mM) in 0.02 M sodium phosphate pH 6.5, 0.1 M NaCl, 0.5 mM DTT, 0.2 mM EDTA, 10% D_2_O. The compounds were dissolved at 100x concentration in deuterated DMSO (DMSO-D_6_) and were added to the NMR samples at 1% (v/v) to give the desired compound concentration (and 1% DMSO-D_6_). Control samples contained APE1 with 1% DMSO-D_6_. For NMR samples that contained MgCl_2_, concentrated APE1 stocks were dialyzed three times against 1 L of NMR buffer that contained 1 ml of a 50% (w/v) slurry of chelex-100 (Bio-Rad), and the buffer used to prepare NMR samples was similarly treated with chelex-100. ^15^N-TROSY experiments were collected at 25°C on a Bruker 800 MHz spectrometer equipped with a room temperature probe, as previously reported [31, 32]. NMR data were processed with NMRPipe [39], and analyzed using NMRFAM-Sparky [40]. The chemical shift perturbation (Δδ) for backbone ^1^H-^15^N resonances (combined) was calculated (by NMRFAM-Sparky) using Eq 2,

$$\Delta \delta ={\left({\left(\Delta \delta_{H}\right)}^{2}+{\left(\Delta \delta_{N}/5\right)}^{2}\right)}^{1/2}$$ (2)

where Δδ_H_ and Δδ_N_ are the perturbations for ^1^H and ^15^N resonances, respectively.

### X-ray crystallography

The samples used for crystallization contained 0.3 mM (10 mg/ml) APE1 in a buffer of 50 mM HEPES pH 7.5, 25 mM NaCl, 1 mM DTT, 5 mM EDTA. Crystals of APE1 (apo form) were grown at room temperature by sitting drop vapor diffusion using 0.5 μl of the APE1 sample and either 0.5 μl or 1.0 μl of mother liquor, which was 0.2 M sodium formate pH 7.0, 20% w/v PEG 3350. For the complex of APE1 and compound **3**, a 2M stock of **3** in DMSO was diluted 20-fold into mother liquor and crystals of apo-APE1 were soaked in this solution for 18 h. Crystals were cryo-protected using the same mother liquor supplemented with 18–20% ethylene glycol, 0.1 M compound **3**, and 5% DMSO prior to flash cooling in liquid nitrogen. We used a high concentration of **3** to increase the probability of success and did not rigorously explore the conditions (compound concentration, soaking time) needed to obtain crystals that would provide high quality diffraction data and yield complete electron density for the compound. The ligand concentrations used for crystal soaking are often higher than that needed to populate the binding site of a protein in aqueous solution. X-ray diffraction data were collected at the Advanced Light Source using beamline 5.0.1 for apo-APE1 and at the Stanford Synchrotron Radiation Lightsource using beamline 12–2 for the APE1-**3** complex. Images were processed using XDS [41] and scaled using Aimless [42] from the CCP4 suite [43] with the autoxds script developed by A Gonzalez and Y Tsai http://smb.slac.stanford.edu/facilities/software/xds. The resolution cutoff was determined based on CC1/2 = 0.3, a standard implementation in Aimless [44]. For our structure of apo APE1 (PDB ID: 7TC3) we used a resolution cutoff of 1.25 Å based on output from Aimless; the electron density map for this model is not significantly different from that observed in a model refined using a lower resolution cutoff of 1.40 Å (S6 Fig). Crystals of apo-APE1 belong to the P2_1_2_1_2 space group; upon soaking crystals of apo-APE1 in mother liquor containing compound **3**, the space group changed to P2_1_. All crystals of the APE1-**3** complex exhibited some degree of translational NCS (which contributes to elevated R-values for this structure). The structures were solved by molecular replacement using Phaser [45] and a previous structure of APE1 as the search model (PDB ID: 4LND). Refinement was performed using BUSTER-TNT [46] or phenix.refine [47] and model building was performed using Coot [48]. The TLS refinement utilized the TLSMD server [49, 50] as described [51]. The crystallographic data (electron density) for the APE1-**3** complex excludes the presence of bound DMSO molecules at any sites, including the two sites for which DMSO was observed in previous APE1 structures (e.g., PDB ID: 6MK3) [52, 53]. PyMOL (http://www.pymol.org) was used to generate structural figures and obtain RMSD values for structural alignments.

## Results and discussion

### NMR studies of the APE1 binding site for indole-2-carboxylic acids

**Fig 2:**
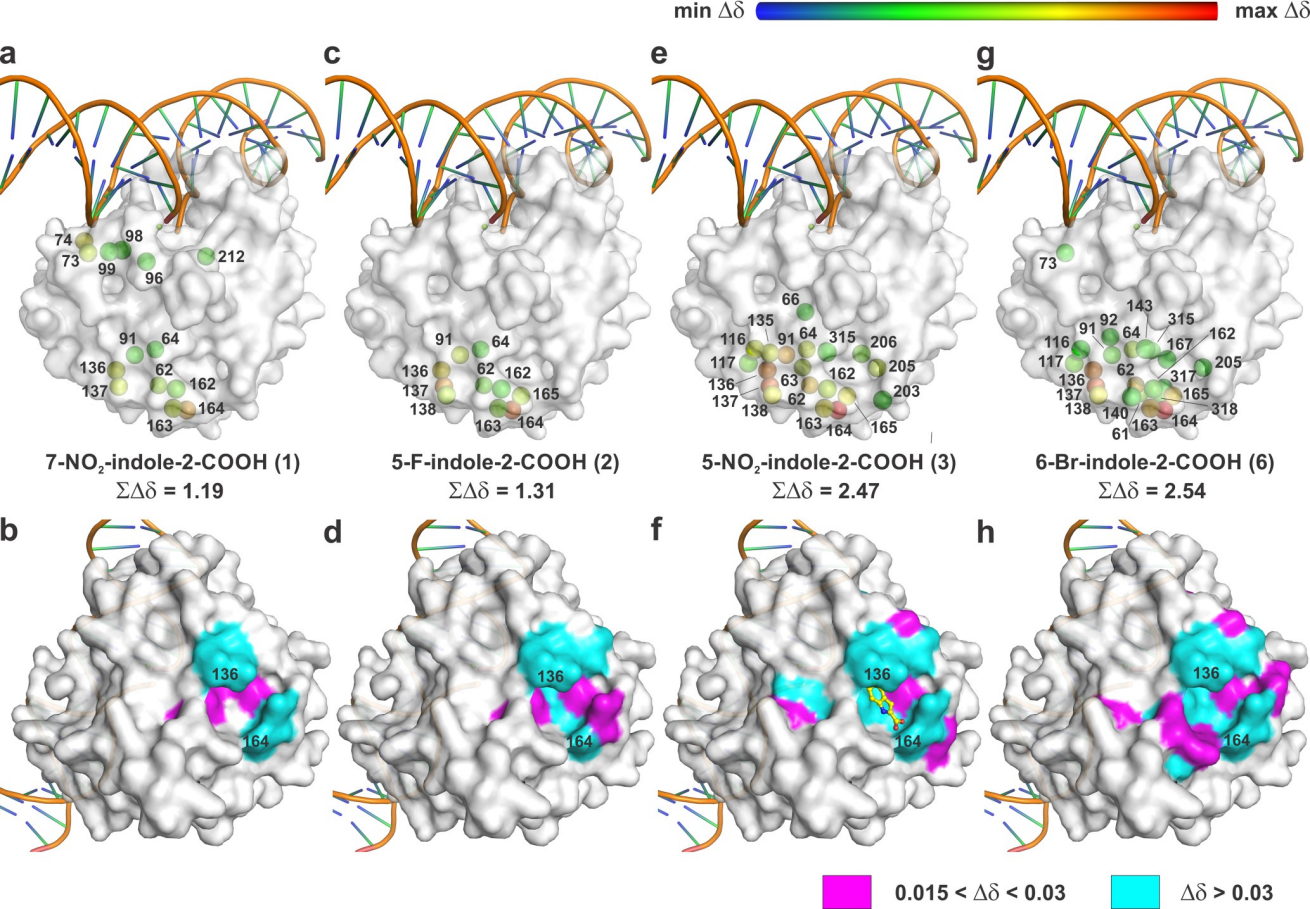
NMR CSPs induced by the indole compounds mapped to a crystal structure of APE1 bound to nicked abasic DNA (enzyme-product complex; PDB ID: 5DFF). In the upper row (a, c, e, g), APE1 is shown in surface format, DNA in cartoon format, and the Mg^2+^ cofactor is shown as a small green sphere, which, together with the nicked site of the DNA, locates the active site. Residues for which the compound induces a CSP (Δδ ≥0.015 ppm) have a sphere centered at the backbone N and colored according to CSP magnitude (log Δδ), with red and blue representing high and low values, respectively (red, Δδ = 0.6; blue, Δδ = 0.001). In the lower row (b, d, f, h), the same APE1 structure is shown in nontransparent surface format and in a different orientation; residues are colored according to CSP magnitude, with cyan for the largest CSPs (Δδ ≥ 0.030 ppm) and magenta for moderate CSPs (0.015 ppm ≤ Δδ < 0.030 ppm). Residues 136 and 164 are labeled, for reference. Shown between each pair of corresponding structures is the compound name and the sum of CSPs (ΣΔδ) induced by that compound for all APE1 residues. For reference, the structure in panel **f** shows compound **3** bound in the remote pocket, as determined by our new crystal structure (Fig 3).

7-nitroindole-2-carboxylic acid or CRT0044876 (**1**, Fig 1) was among the first reported APE1 inhibitors [54]. It has been widely used in studies as an inhibitor of APE1 or the BER pathway overall [55–57] and it is marketed for this purpose by multiple vendors. CRT0044876 was reported to inhibit APE1 with an IC_50_ of 3 μM and sensitize cells to antitumor agents [54]. A similar compound, 5-fluoroindole-2-carboxylic acid (**2**), was reported to inhibit APE1 with an IC_50_ of 10 μM [58]. Given their small size and reported potency, we sought to determine the APE1 binding site of compounds **1** and **2** using NMR chemical shift perturbation (CSP) experiments. Previous studies show the power of this NMR approach for human APE1, which has nicely resolved backbone ^1^H-^15^N resonances in 2D ^15^N-TROSY spectra [31, 32]. We reported chemical shift assignments for >90% of the backbone (^1^H, ^15^N, ^13^Cα, ^13^CO) and ^13^Cβ resonances for apo (Mg^2+^-free) APE1^ΔN38^, a construct lacking the N-terminal region that is disordered and dispensable for endonuclease activity [34–36]. Here, we collected ^15^N-TROSY spectra for apo APE1^ΔN38^ (0.15 mM) in the absence and presence of indole compounds (1 mM). We determined the CSP (Δδ) for backbone ^1^H-^15^N resonances of assigned residues, where Δδ is for ^1^H and ^15^N combined (S1–S4 Figs). The NMR results are illustrated in Fig 2, where residues of APE1 for which an indole compound induces significant CSPs (Δδ ≥ 0.015 ppm) are indicated on a structure of the enzyme bound to nicked abasic DNA (an enzyme-product complex; PDB ID: 5DFF) [59]. In the upper row of structures shown in this figure, the perturbed residues are denoted by a sphere that is centered at the backbone N and colored according to CSP magnitude, with red and blue representing high and low values, respectively. In the lower row, a nontransparent surface representation of the same APE1 structure is shown, in a different orientation, with residues exhibiting the largest CSPs (Δδ ≥ 0.030 ppm) colored cyan and those exhibiting moderate CSPs (0.015 ppm ≤ Δδ < 0.030 ppm) colored magenta. Shown between each pair of corresponding structures is the compound name and the sum of CSPs (ΣΔδ) induced by that compound for all assigned residues of APE1.

Results for compound **1** reveal substantial CSPs (Δδ ≥ 0.015 ppm) for 14 backbone ^1^H-^15^N pairs, six located in the DNA-binding groove and eight clustered together at a pocket that is distal from the active site (Fig 2A and 2B). This remote pocket includes residues R136, Q137 and S164, which exhibit some of the largest CSPs (S1 Fig). Compound **2** induces CSPs (Δδ ≥ 0.015 ppm) for ten residues, all clustered at the remote pocket that includes residues R136, Q137 and S164, which exhibit the largest CSPs (Figs 2C and 2D and S2). Given these intriguing findings, we performed NMR studies on other indole-2-carboxylic acids and found two compounds that induce a large number of CSPs. 5-nitroindole-2-carboxylic acid (**3**) generates CSPs for 20 residues, all clustered near the remote Q137-S164 site, 14 of which exhibit relatively strong CSPs, with Δδ ≥0.03 ppm (Figs 2E and 2F and S3). 6-bromoindole-2-carboxylic acid (**4**) induces substantial CSPs for 22 residues, all but one located near the remote pocket (Q137-S164) and eight residues exhibit relatively strong CSPs (Figs 2G and 2H and S4). Given that previous crystal structures show DMSO can bind the APE1 surface, at least when APE1 crystals are soaked in solution containing 5% DMSO, we investigated DMSO binding under the NMR conditions. We find that DMSO, at a concentration of 1%, causes negligible CSPs, with only three residues that exhibit Δδ >0.015 and none with Δδ >0.017 ppm (S5 Fig). Thus, our NMR results show, unexpectedly, that indole-2-carboxylic acids target a remote APE1 pocket that includes residues R136, Q137, and S164 (among others) and is distal from the active site.

### Crystal structure of 5-nitroindole-2-carboxylic acid bound to APE1

**Fig 3:**
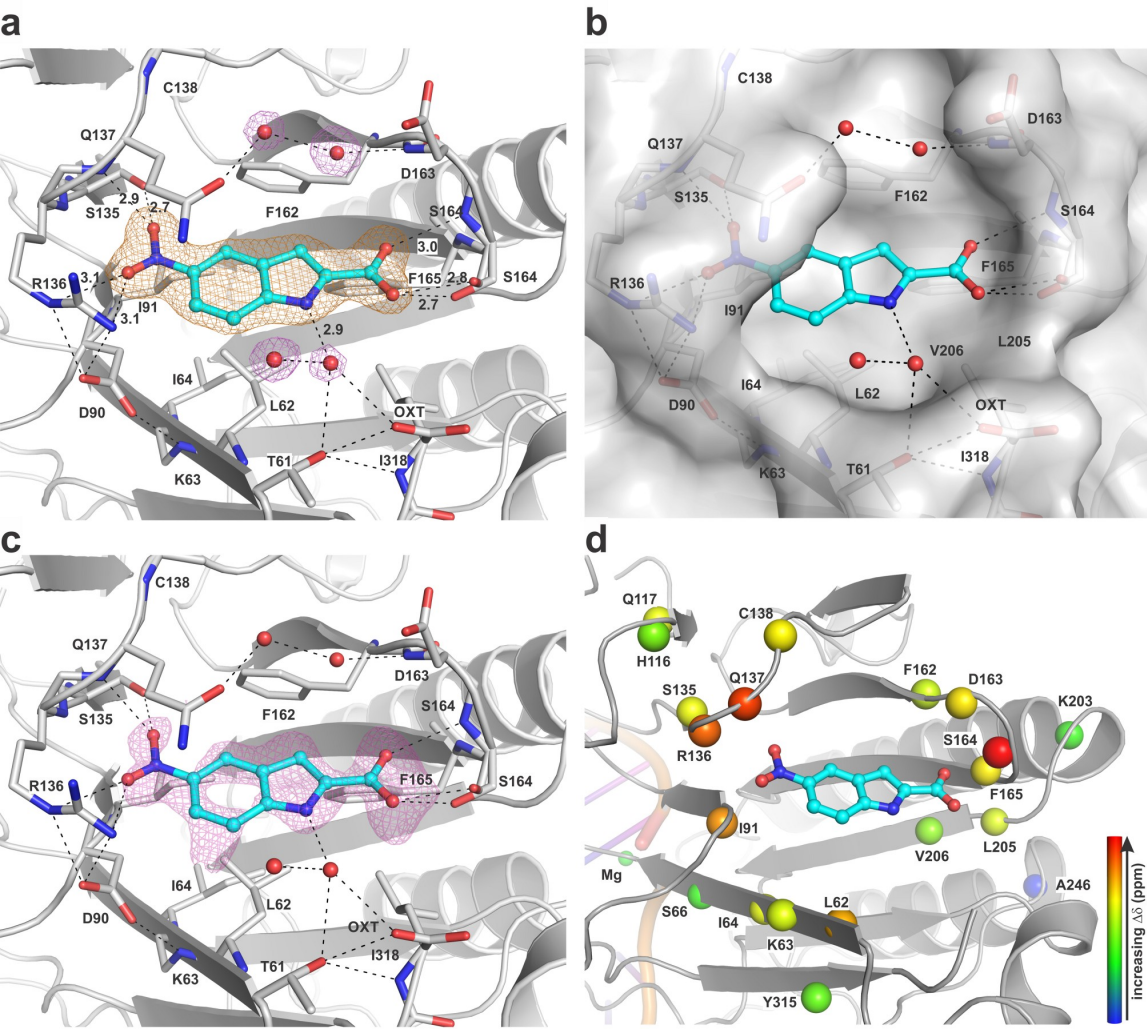
Structure of APE1 with 5-nitroindole-2-carboxylic acid (3) bound at a remote site. (a) Close-up view of **3** (cyan) bound to APE1 (cartoon, some side chains in stick format). The 2*F*_o_-*F*_c_ electron density map, contoured at 1.0 *σ*, is shown for **3** and some water molecules. Dashed lines represent hydrogen bonds with distances shown for those involving compound **3**. (b) Same view with a surface rendering of APE1 to further illustrate the binding pocket. (c) Same view as in panel a but showing the *F*_o_-*F*_c_ electron density map (difference map), contoured at 2.2 *σ*, for a model that lacks compound **3**. (d) Same view as in panel a, with spheres at the backbone N of residues exhibiting NMR CSPs (Δδ ≥0.015 ppm). Sphere color reflects CSP magnitude (log Δδ), with red and blue representing high and low CSPs, respectively. DNA and Mg^2+^ in the background were modeled in by aligning our structure (PDB ID: 7TC2) with a previous structure of DNA-bound APE1 (PDB ID: 5DFF).

We also sought to obtain a crystal structure of APE1 in complex with one of the indole compounds and found success with 5-nitroindole-2-carboxylic acid (**3**). We soaked **3** into crystals of apo APE1 and solved a high-quality structure at 1.43 Å resolution (S1 Table; PDB ID 7TC2). Compound **3** features strong electron density, defining its pose and binding interactions with the enzyme (Fig 3A and 3B). Additional evidence for binding of compound **3** to this remote pocket is provided by the *F*_o_-*F*_c_ map calculated using a model that does not include the compound (Fig 3C) The 5-nitro of **3** accepts hydrogen bonds from S135 and R136 and the backbone N of Q137, and the 2-carboxyl of **3** is contacted by the hydroxyl and the backbone N of S164. The pocket features many hydrophobic residues, including L62, I64, I91, F162, and F165. Together, these nine residues are among the 11 that exhibit the largest NMR CSPs (Δδ ≥ 0.03 ppm) induced by **3**, demonstrating excellent agreement between the crystallographic and NMR results (Fig 3D). The NMR results suggest the three other indole compounds bind the same site, though the detailed interactions will vary with indole functional groups. Notably, the remote binding site revealed by our experimental findings is consistent with a site predicted using computational approaches [26]. A recent paper reports that multiple crystal structures have been determined for APE1 with various small molecules (fragments) bound at the endonuclease site or a secondary site, although details are not provided regarding the ligand(s), the binding site(s), or the potential effect of the fragments on APE1 activity [60].

To evaluate APE1 structural changes associated with binding to compound **3**, we solved a high resolution (1.25 Å) structure of human APE1 in its apo form, that is, without any metal in its Mg^2+^-binding site (S1 Table; PDB ID: 7TC3). Notably, this is the first such structure reported for wild-type human APE1. While two structures were previously solved for the apo form of human APE1 (PDB ID 4QHD, 6MK3) [52, 53] they carry a mutation (C138A) for a residue in the remote pocket that binds the indole-2-carboxylic acids, prompting us to solve a structure of wild-type enzyme. Superposition of the structures for these apo forms of human APE1 (wild-type and C138A) indicates that the overall conformations are very similar, with RMSD values of 0.100 Å for backbone Cα atoms and 0.247 Å for all non-hydrogen atoms, with minor structural differences in the remote binding pocket near C138 (S6 Fig). Regarding APE1 conformational changes caused by the binding of compound **3**, superposition of our two new structures for apo APE1, free and in complex with compound **3**, reveals that binding of **3** induces minor changes in overall structure with some significant changes in the remote binding site for residues including L62, D90, R136, Q137, F162, S164, F165, and L318 (S7 Fig).

### Colloidal aggregation of indole-2-carboxylic acids

**Fig 4:**
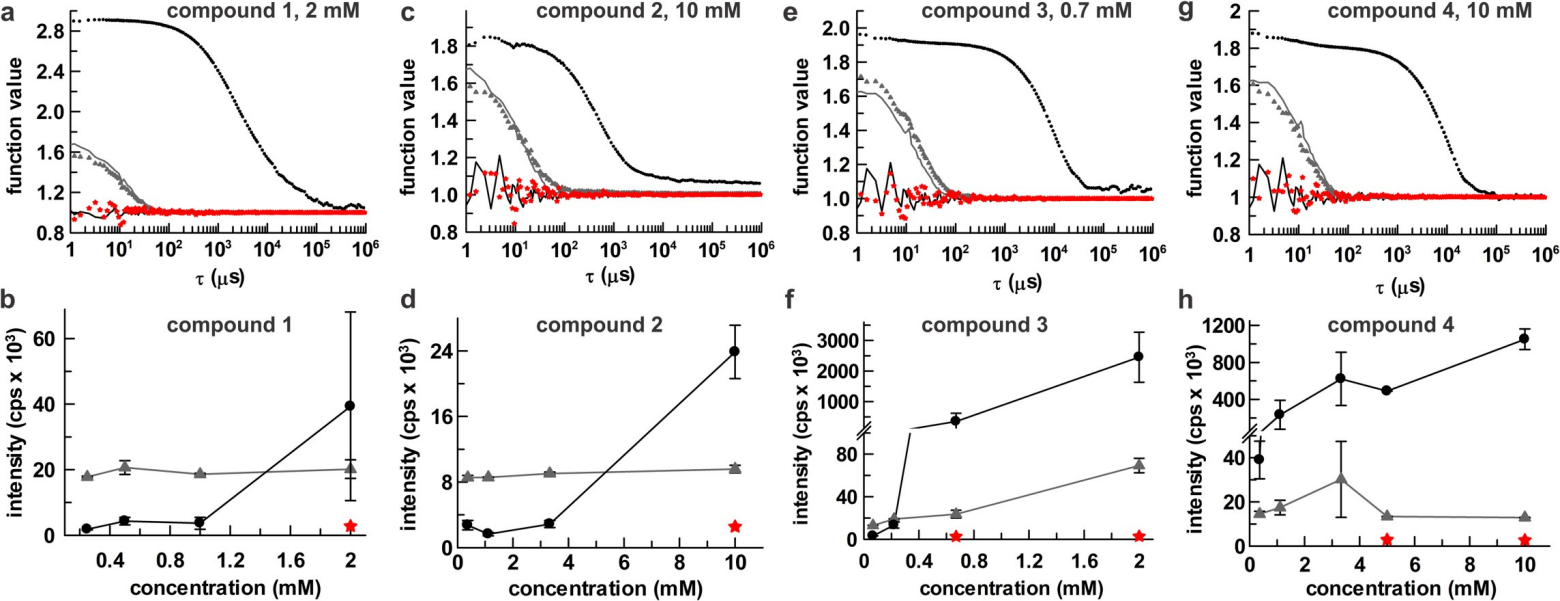
Aggregation of the four indole compounds (1–4) studied by dynamic light scattering (DLS). Panels a, c, e, and g show autocorrelation functions for the compound in buffer that lacks (circle) or contains (triangle) detergent (0.05% Brij 35). Solid lines are controls for buffer alone (no compound) in the absence (black) or presence (grey) of detergent. Data for compounds in detergent-free buffer after centrifugation are shown as red stars. Panels b, d, f, and h show scattering intensity (kilocounts per second) as a function of compound concentration for samples that lack detergent (circles) or contain detergent (triangles). Data for the compound in detergent-free buffer after centrifugation is indicated by red stars. In all cases, compounds were introduced from 100x stocks in DMSO; compound-free samples contained 1% DMSO.

Intrigued by our findings that indole-2-carboxylic acids target a remote pocket of APE1, and the potential implications for allosteric inhibition, we sought to revisit prior reports that two of the compounds (**1**, **2**) inhibit APE1 with low micromolar IC_50_ and to test inhibition by the others (**3**, **4**). However, we first investigated whether these compounds form colloidal aggregates, which could potentially sequester APE1 and cause non-specific inhibition [28, 29]. A vast number of small molecules, including approved drugs, have been shown to exhibit such artifactual inhibition, and this is a major cause of false positives in screening campaigns [30]. Using dynamic light scattering (DLS), a prominent method for studying compound aggregation, we find that compound **1** forms colloidal aggregates at a concentration of 2 mM, as indicated by the autocorrelation function (Fig 4A) and scattering intensity (Fig 4B) for a sample containing the compound relative to a control (DMSO). It is well established that compound aggregation can be disrupted by non-ionic detergents (e.g., Triton X-100, Tween-20, among others) [29, 61, 62], and DLS results show that aggregation of **1** is disrupted by detergent (0.05% Brij 35). Moreover, we find that aggregates of **1** can be pelleted out of solution by centrifugation (60 min, 16000*g*), consistent with findings for other compounds that are known to form aggregates [29]. The DLS results indicate that **2** is free of aggregates at concentrations below 3 mM (Fig 4C and 4D). While **2** forms aggregates at a concentration of 10 mM, this is disrupted by detergent and the aggregates are pelleted by centrifugation. Of the four indole compounds examined, **3** appears most prone to aggregation, forming aggregates at or above 0.7 mM (Fig 4E and 4F). Aggregation of **3** is disrupted by detergent and aggregates are pelleted by centrifugation. Compound **4** forms aggregates at concentrations of 1 mM or higher (Fig 4G and 4H); aggregation is disrupted by detergent and the aggregates can be pelleted by centrifugation.

While three of the indoles (**1**, **2**, **4**) do not aggregate at the concentration used for the NMR studies (1 mM), compound **3** aggregates at a concentration (0.7 mM) similar to that used for NMR. However, the NMR spectra for APE1 shows that the addition of compound **3** does not cause a substantial decrease in peak intensity, indicating that APE1 remains predominantly free in solution rather than bound to large compound aggregates (which would greatly suppress peak intensity). Observation that **3** induces substantial CSPs indicates that some fraction of the compound remains monomeric and available to bind APE1. These results are not unexpected; given the high concentration of APE1 in the NMR sample, a relatively small fraction of the protein could potentially saturate the surface of compound aggregates that form under the NMR conditions [63]. The crystals used to determine the structure of APE1 in complex with **3** were generated by soaking preformed crystals of apo APE1 in a solution of mother liquor with 5% DMSO and 100 mM compound **3**. While this concentration of **3** is two orders of magnitude above that observed to form aggregates, our result indicates that some fraction of the compound, perhaps a small fraction, is populated in the monomeric form under the crystallization conditions.

### Weak inhibition of APE1 by indole-2-carboxylic acids

**Fig 5:**
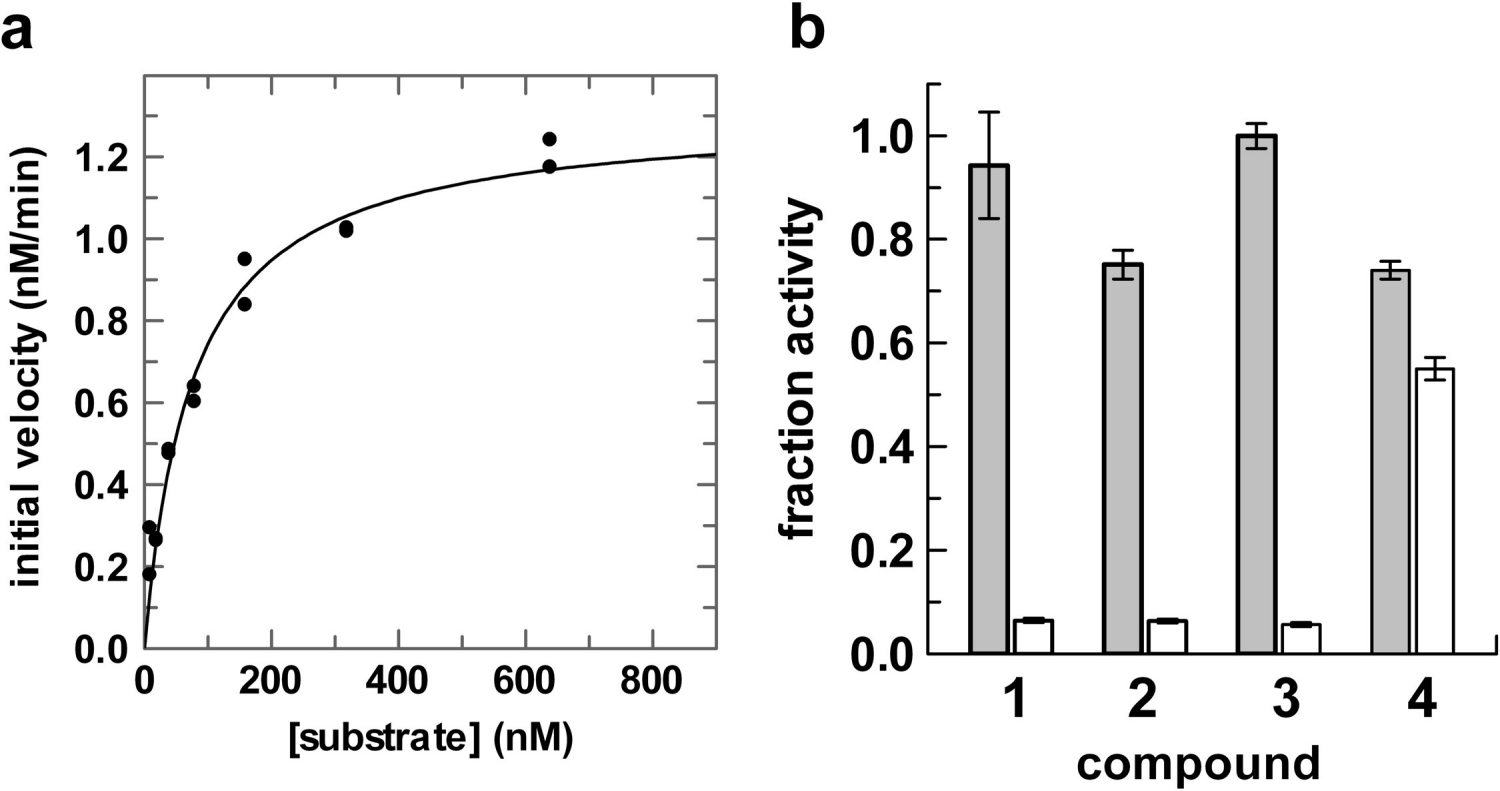
Michaelis-Menten parameters for APE1 endonuclease activity and inhibition by compounds 1–4. (a) Fitting of initial velocity versus substrate concentration gives *k*_cat_ = 2.2 ± 0.1 s^−1^ and *K*_m_ = 76 ± 9 nM. Reactions were performed at room temperature (23°C) with 10 pM APE1. (b) Fraction activity of APE1 in the presence of compound **1** (2 mM), **2** (10 mM), **3** (0.7 mM), or **4** (10 mM) in reactions that included 0.05% Brij 35 and 0.1 mg/ml BSA (grey bars) or 100-fold lower concentrations of Brij 35 and BSA (white bars). Fraction activity (FA) is defined as FA = *v*_0_^cpd^/*v*_0_^DMSO^, where *v*_0_^cpd^ and *v*_0_^DMSO^ are initial velocities for reactions that contain or lack a compound, respectively. All samples, with or without compound, contained 1% DMSO.

We next sought to characterize APE1 inhibition for the indole compounds (**1**–**4**) under conditions that reduce compound aggregation, using a reaction buffer that contained detergent (0.05% Brij 35). The buffer also contained bovine serum albumin (BSA) at a concentration of 0.1 mg/ml (1.5 μM), which could potentially bind to residual compound aggregates and suppress binding of APE1, which is present at a much (100,000-fold) lower concentration than BSA [63]. We employed an HPLC assay similar to our method for monitoring DNA glycosylase activity [37], where DNA fragments generated by APE1 cleavage at abasic sites are resolved by anion exchange under denaturing (pH 12) conditions. Using this assay, we determined the initial velocity (*v*_0_) at varying substrate concentrations and fitted the dependence of *v*_0_ on [S] to obtain the steady-state (Michaelis-Menten) parameters *k*_cat_ = 2.2 ± 0.1 s^−1^ and *K*_m_ = 76 ± 9 nM (Fig 5A). The *K*_m_ observed here is in the range of values reported in prior studies (28 nM to 100 nM) for which the parameter was carefully determined under similar experimental conditions [64–66].

We investigated APE1 inhibition for the indole compounds at the highest concentration that lacked aggregation in the presence of detergent (0.05% Brij35), as indicated by the DLS studies above, including **1** at 2 mM, **2** and **4** at 10 mM, and **3** at 0.7 mM (Fig 5B). Even at these high concentrations, inhibition was weak or not detected as shown by fraction activity (FA) ranging from 0.75 to 1.0. (FA = *v*_0_^cpd^/*v*_0_^DMSO^, where *v*_0_^cpd^ and *v*_0_^DMSO^ are initial velocities for reactions that contain or lack a compound, respectively). We also investigated the effect of these compounds on APE1 activity in a reaction buffer that contained a 100-fold lower concentration of detergent (0.0005% Brij35) and BSA (0.001 mg/ml) and find that FA ranges from 0.06 to 0.55 (Fig 5B). Observation that apparent inhibition is stronger in the absence of detergent (and BSA) supports a non-specific mechanism whereby the enzyme is sequestered by compound aggregates [29, 62]. Our results do not support the prior findings that **1** and **2** inhibit APE1 with an IC_50_ of 10 μM or below [54, 58]. Notably, activity assays in the original work for **1** and **2** were reported to have been performed in the absence of detergent or BSA, and at a higher temperature (37°C) than used in our studies (23°C). These conditions could render APE1 more susceptible to non-specific inhibition through compound aggregation [67].

Together, our NMR and structural studies demonstrate that the indole-2-carboxylic acids target a remote pocket of APE1 (Q137-S164). However, the activity assays show that these compounds inhibit APE1 only under conditions that permit compound aggregation and not under conditions that disrupt it (with detergent). These results lead to the conclusion that binding of indole-2-carboxylic acids to the newly identified remote binding pocket does not substantially alter the repair activity of APE1. Additional studies will be needed to determine whether binding of other compounds at the remote site can modulate APE1 repair activity in an allosteric manner.

### Characterization of other reported APE1 inhibitors

**Fig 6:**
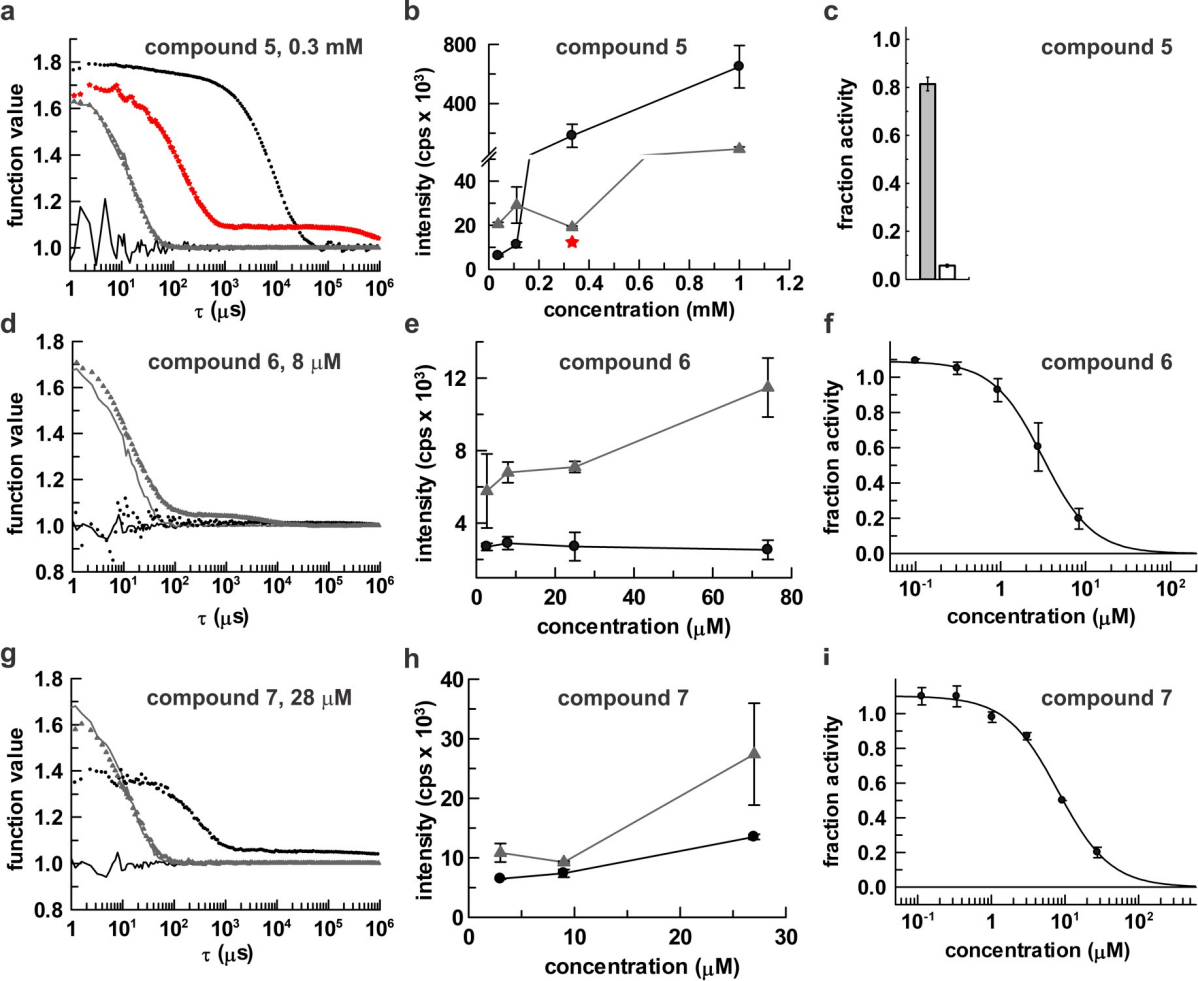
Evaluation of compounds 5, 6, and 7 for aggregation by DLS and for inhibition of APE1. Autocorrelation functions from DLS are shown in panels a, d, and g for the compound in buffer that lacks (circle) or contains (triangle) detergent (0.05% Brij 35). Solid lines are controls for buffer alone (no compound) in the absence (black) or presence (grey) of detergent. Data for compounds in detergent-free buffer after centrifugation is indicated by red stars. Panels b, e, and h show scattering intensity (kilocounts per second) versus compound concentration for compounds in buffer that lacks (circles) or contains (triangles) detergent. Data for compounds in detergent-free buffer after centrifugation is indicated by red stars. Panels c, f, and i show fraction activity of APE1 in the presence of a given compound. Data for **5** are shown at a single concentration of 0.33 mM for reactions that included Brij 35 (0.05%) and BSA (0.1 mg/ml) (grey bar) or 100-fold lower concentrations of these components (white bar). The dependence of FA on compound concentration gives an IC_50_ of 3.1 ± 0.3 μM and slope of 1.5 ± 0.2 for **6**, and an IC_50_ of 8.1 ± 0.6 μM and slope of 1.2 ± 0.1 for **7**. Compounds were introduced to DLS samples or enzyme reactions from 100x stocks in DMSO, and compound-free control samples also contained 1% DMSO.

We also investigated three other compounds that were previously found to inhibit APE1 *in vitro* and in human cells (**5**, **6**, **7**; Fig 1) [66, 68, 69]. These compounds were selected because they are considered to be among the most prominent inhibitors emerging from several compound screening studies, as judged by their coverage in the literature [27, 66, 68–71]. In addition, compound **7** is sold by multiple vendors as an inhibitor of APE1 (referred to as APE1 inhibitor III). We examined the propensity of these compounds to form aggregates and their ability to inhibit APE1 under conditions that disrupt aggregation. Compound **5**, or myricetin, was identified as an APE1 inhibitor through screening a collection of 1280 drug like molecules (LOPAC^1280^) [68]. We find that **5** aggregates at concentrations of 0.33 mM and above. Aggregation of **5** is disrupted by detergent (0.05% Brij35) and the aggregates can be pelleted by centrifugation (Fig 6A and 6B). Notably, another study found that myricetin forms aggregates, that the aggregates are disrupted by detergent, and that it inhibits enzymes nonspecifically [72]. Myricetin exhibits weak inhibition of APE1 at a concentration of 0.33 mM (Fig 6C), the highest concentration found to lack aggregation in the presence of detergent. As such, it was not feasible to determine an IC_50_. Notably, in the absence of detergent and BSA, APE1 activity is greatly impaired by myricetin at the same concentration (0.33 mM, Fig 6C). Together, these observations suggest the previous finding that myricetin inhibits APE1 with an IC_50_ of 0.3 μM could likely be explained by nonspecific inhibition through compound aggregation [68].

Compound **6** was identified as an APE1 inhibitor through a screen of 60000 compounds, and it was found to inhibit incision of AP sites in cell extracts and repair of AP sites in glioblastoma cells (SF767), and to potentiate the cytotoxicity of MMS and TMZ [69]. We characterized APE1 inhibition by compound **6**, often termed AR03 (APE1 repair inhibitor 3), finding an IC_50_ of 3.7 ± 0.3 μM (Fig 6F). While the reactions were performed with detergent, our DLS results indicate that in the absence of detergent, AR03 does not aggregate at the concentrations used to determine the IC_50_ (Fig 6D and 6E). Our results are consistent with the previous finding that AR03 inhibits APE1 *in vitro* with an IC_50_ of 2 μM [69].

Compound **7**, also known as APE1 Inhibitor III or MLS000419194, was identified through a screen of the NIH Molecular Libraries Small Molecule Repository (MLSMR) and it was found to inhibit AP site incision in HeLa cell extracts, potentiate the cytotoxicity of MMS and TMZ, and enhance the abundance of AP sites in MMS treated cells [66, 70]. However, a recent study concluded that the toxicity of **7** involves off-target effects, based on findings that for two mammalian cell lines which are viable upon knockout of APE1 (HEK293 FT, CH12F3), the toxicity of **7** was as high (or higher) in APE1-deficient cells relative to APE1 proficient counterparts [71]. We find that APE1 is inhibited by compound **7** with an IC_50_ of 8.1 ± 0.6 μM (Fig 6I). While the reactions were performed with detergent, DLS studies indicate that **7** does not aggregate in the absence of detergent at the range of concentrations used to obtain the IC_50_ (Fig 6G and 6H). Our result is consistent with prior findings that **7** inhibits APE1 activity *in vitro* with an IC_50_ ranging from 2 to 14 μM [66, 70]. However, the report noted above that the toxicity of **7** involves off-target effects, together with our NMR results below, suggest that the observed inhibition of APE1 is unlikely to involve specific binding of **7** to APE1.

### Compounds 6 and 7 do not bind specifically to apo-APE1

We used NMR CSP experiments to investigate the binding of compounds **6** and **7** to APE1. The ^15^N-TROSY spectra for apo APE1^ΔN38^ in the absence and presence of compound, and a plot of CSP (Δδ) versus amino acid residue are shown in S8 and S9 Figs. Compared to the CSPs induced by the indole compounds (**1**–**4**, above), the CSPs for **6** are weak, with no residues exhibiting Δδ >0.015 ppm. The experiments were performed using 0.10 mM APE1 and 0.30 mM of compound **6**, which should give a saturating concentration of the compound if the IC_50_ of 0.003 mM observed here even roughly approximates its dissociation constant (*K*_d_). Thus, the NMR CSPs provide no evidence that compound **6** binds specifically to apo-APE1.

The NMR results also reveal that compound **7** induces weak CSPs for apo APE1, with only two residues exhibiting Δδ >0.015 ppm and none with Δδ >0.017 ppm. The NMR sample contained 0.03 mM compound **7** and 0.05 mM APE1, giving a molar ratio of 0.6. These conditions were used because **7** was found to precipitate at higher concentrations (≥0.09 mM) in NMR samples. To minimize precipitation of **7**, the NMR samples (including DMSO control) contained detergent (0.05% Brij 35) at the same concentration used for the activity assays. Additional controls showed that detergent itself does not generate CSPs for APE1 (not shown). Thus, the NMR CSP studies provide no evidence that compound **7** binds specifically to apo-APE1. Previous studies, using electrophoretic mobility shift assays (EMSAs) in a buffer that lacked Mg^2+^, showed that a relatively tight enzyme-substrate (ES) complex (*K*_d_ < ~5 nM) involving APE1 (28 nM) and AP-DNA (10 nM) could be disrupted by compound **7** [66]. In particular, the population of AP-DNA bound was reduced from 100% (absence of **7**) to about 30% and 5% in the presence of **7** at a concentration of 0.01 mM and 0.03 mM, respectively, indicating **7** binds with a *K*_d_ <0.01 mM to either APE1 or to AP-DNA. The absence of a detectible interaction between apo-APE1 (0.05 mM) and compound **7** (0.03 mM) in our NMR studies indicates that if **7** binds to apo-APE1, the interaction is relatively weak (*K*_d_ >> 0.03 mM), suggesting the previous EMSA results likely reflect binding of **7** to AP-DNA rather than APE1.

### Binding of Mg^2+^ causes extensive NMR perturbations for APE1

**Fig 7:**
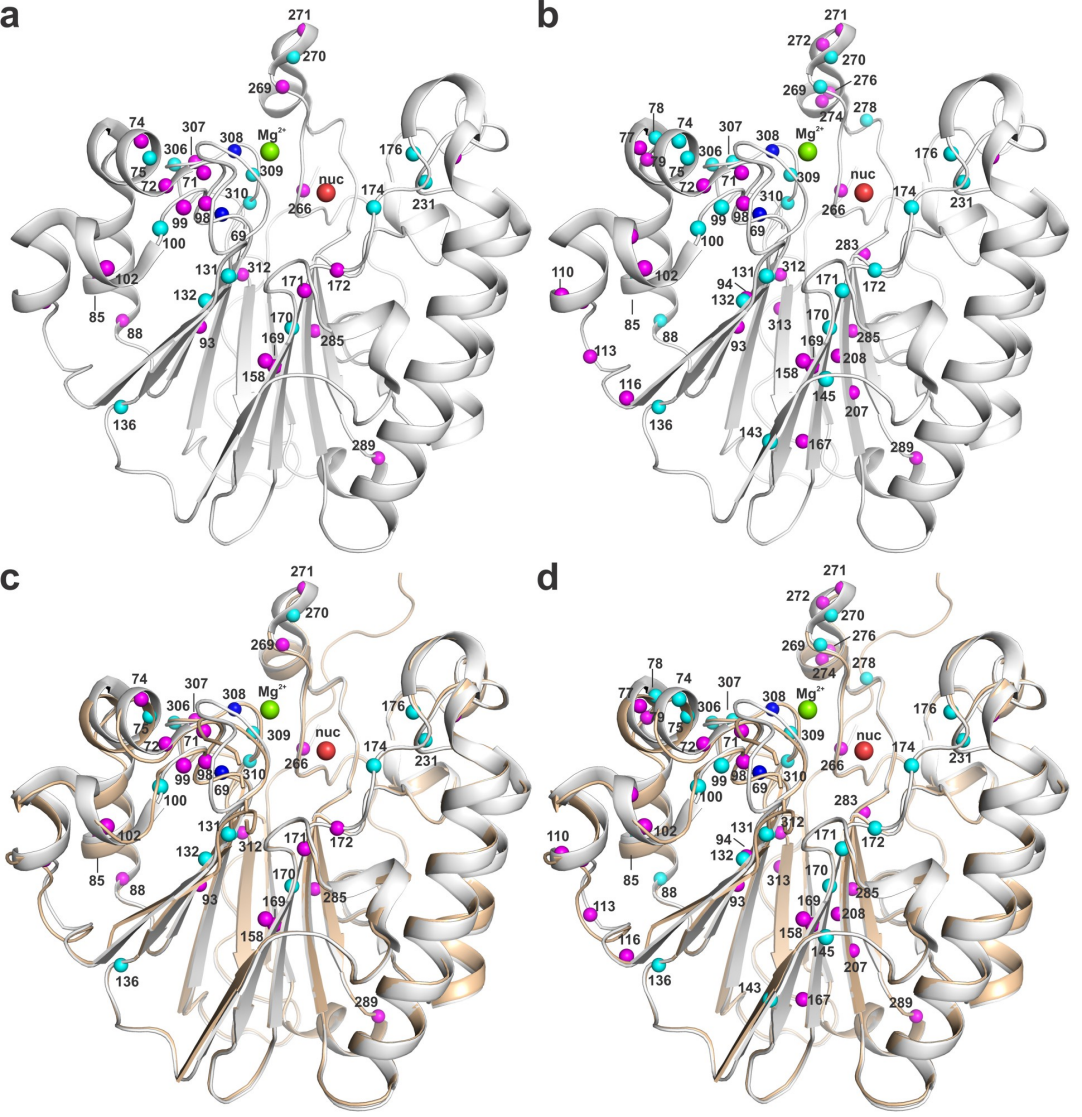
Mg^2+^-induced NMR chemical shift perturbations shown on a structure of APE1. Residues of APE1 that exhibit NMR chemical shift perturbations induced by the presence of MgCl_2_ at a concentration of (a) 0.25 mM or (b) 1.0 mM are indicated by spheres (at backbone N) on a structure of Mg^2+^-bound human APE1 (PDB ID 4LND). Sphere color reflects CSP magnitude, with magenta for Δδ of 0.015 to 0.030 ppm and cyan for Δδ ≥ 0.030 ppm. The Mg^2+^ cofactor is shown in as a green sphere and the putative nucleophilic water molecule is a red sphere (“nuc”). The NMR spectra and CSPs are shown in S10 and S11 Figs. (c, d) Superposition of structures for human APE1 in the apo state (tan, PDB ID: 7TC3 as reported here) and in the Mg^2+^-bound state (white; PDB ID 4LND) with spheres for residues that experience CSPs upon binding Mg^2+^ at a concentration of (**c**) 0.25 mM or (**d**) 1.0 mM. Sphere coloring and position are as described for panels a and b.

Given the findings above, we sought to investigate whether compounds **6** or **7** might bind to the Mg^2+^-bound form of APE1. To enable these studies, we collected ^15^N-TROSY spectra for APE1^ΔN38^ (0.10 mM) in the presence varying concentrations of MgCl_2_ (0.063 mM, 0.125 mM, 0.25 mM, 0.50 mM, 0.75 mM, 1.0 mM), such that we could monitor the progression of backbone ^1^H-^15^N resonances perturbed by binding of Mg^2+^. Crystal structures of Mg^2+^-bound APE1 have been determined, using crystals grown from a sample of APE1 with 1.0 mM MgCl_2_, a concentration that is sufficient to fully populate the metal-binding site with Mg^2+^ [52, 73]. We find that a concentration of 0.25 mM MgCl_2_ causes substantial NMR perturbations (Δδ ≥0.015 ppm) for 35 residues, with 13 of the residues exhibiting relatively large CSPs (Δδ ≥0.030 ppm) (S10 Fig). Moreover, increasing the MgCl_2_ concentration to 1.0 mM causes perturbations for 54 residues (Δδ ≥0.015 ppm), including 23 CSPs that are relatively large (Δδ ≥0.030 ppm) (S11 Fig). The residues perturbed by Mg^2+^ are largely clustered around the Mg^2+^-binding site, as defined by structures of Mg^2+^-bound APE1 [52, 73]. To illustrate this point, residues that exhibit Mg^2+^-induced CSPs are marked on a structure of Mg^2+^-bound APE1, with magenta spheres reflecting moderate CSPs (Δδ of 0.015 to 0.030 ppm) and cyan spheres denoting larger CSPs (Δδ ≥0.030 ppm) (Fig 7A and 7B). A superposition of our new structure for apo-APE1 and a prior structure of Mg^2+^-bound APE1 illustrates that binding of Mg^2+^ causes substantial conformational changes in APE1, most near the Mg^2+^ site, as expected (Fig 7C and 7D). The Mg^2+^-induced NMR shift perturbations reflect these conformational changes, in addition to changes in the chemical environment near the Mg^2+^-binding site. We note that NMR perturbation data are provided for APE1 with two concentrations of MgCl_2_ (0.25 mM, 1.0 mM) because we observed that for [MgCl_2_] >0.25 mM, some peaks in the TROSY spectra become weak (e.g., residues 170, 172) or disappear (residues 100, 131, 309) (S10 and S11 Figs). As such, we also used two MgCl_2_ concentrations to monitor binding of compounds **6** and **7** to the Mg^2+^-bound form of APE1.

### Compounds 6 and 7 do not bind specifically to Mg^2+^-bound APE1

We performed ^15^N-TROSY experiments to investigate whether compounds **6** or **7** bind to the Mg^2+^-bound form of APE1. We find that compound **6** (0.30 mM) does not induce CSPs (Δδ >0.015 ppm) for any residue of APE1 (0.10 mM) in the presence of MgCl_2_ at a concentration of 0.25 mM or 1.0 mM (S12 Fig). Given our findings that the indole-2-carboxylic acids, and the simple Mg^2+^ ion, cause large CSPs for many residues of APE1, the NMR results for compound **6** provide no evidence that it binds specifically to Mg^2+^-APE1, similar to findings above for apo APE1. It was reported that compound **6** has some affinity for binding to DNA [69], which could potentially account for the observation that it inhibits APE1 *in vitro*. We cannot rule out the possibility that **6** inhibits APE1 by binding selectively to the enzyme-substrate complex (uncompetitive inhibition), though this seems unlikely.

NMR studies reveal that compound **7** (0.030 mM) also fails to induce CSPs (Δδ >0.015 ppm) for any backbone residue of APE1 (0.05 mM) in the presence of MgCl_2_ at a concentration of 0.25 mM or 1.0 mM (S13 Fig). Together, our NMR results indicate that **7** does not bind specifically to Mg^2+^-APE1 or apo APE1. Thus, our findings are not consistent with the suggestion that compound **7** inhibits APE1 by binding to the active site [66, 70]. Observation that **7** inhibits APE1 *in vitro* could potentially reflect binding of **7** to DNA (nonspecific) or to AP-DNA. As noted above, such a mechanism could potentially account for findings (by EMSA) that compound **7**, at a concentration of 0.03 mM (as used in our NMR studies), nearly completely disrupts a tight ES complex involving APE1 (28 nM) and AP-DNA (10 nM) [66]. Notably, binding of compound **7** to AP-DNA could potentially explain findings that its toxicity in mammalian cells involves substantial off-target effects [71].

## Conclusions

We employed structural, biophysical, and biochemical approaches to characterize several compounds previously reported to inhibit APE1. CRT0044876 (**1**), the first reported inhibitor, has been used in many studies to target APE1 or the BER pathway overall, and it is offered for this purpose by multiple vendors. Our findings indicate that CRT0044876 forms aggregates and is a weak inhibitor of APE1 under conditions that disrupt compound aggregation. Similar findings were obtained for three similar indole-2-carboxylic acids, one of which (**2**) was also reported to inhibit APE1. Our results suggest that prior findings of APE1 inhibition by **1** and **2** (micromolar IC_50_) could be explained by non-specific inhibition through compound aggregation. Remarkably, our results also show that the indole compounds bind at a pocket of APE1 that is distal from its active site, with specific interactions defined in a crystal structure of APE1 in complex with compound **3**. While the possibility of discovering allosteric inhibitors that target this site is alluring, our findings that the indoles bind the remote site but lack APE1 inhibition raise questions about the potential effectiveness of such an approach. Nevertheless, our results do not exclude the possibility that other compounds might allosterically modulate APE1 activity by targeting this remote site. Our results also show that myricetin (**5**) forms colloidal aggregates and is a poor inhibitor of APE1 under conditions that disrupt compound aggregation. Our studies of two other reported APE1 inhibitors (**6**, **7**) give IC_50_ values in the low micromolar range, in agreement with previous findings. However, NMR studies performed for APE1 in the apo- and Mg^2+^-bound states show that compounds **6** and **7** do not cause substantial CSPs for any backbone residues of APE1. Thus, the NMR results provide no evidence that these compounds bind specifically to APE1. Our findings for these previously reported APE1 inhibitors should help guide decisions regarding their use in future studies of APE1 and inform future efforts to develop novel inhibitors of this important BER enzyme.

## Supporting information

S1 Fig APE1 chemical shift perturbations induced by 7-nitroindole-2-carboxylic acid (1). (a) ^15^N-TROSY spectra for APE1 (0.15 mM) in the absence (black) or presence (red) of **1** (1 mM). (b) Bar chart of chemical shift perturbations (Δδ) for backbone ^1^H, ^15^N resonances (combined) versus amino acid residue. Dashed lines are shown at Δδ values of 0.015 and 0.030. Residues exhibiting Δδ ≥0.015 are labeled in both figures.
(TIF)

S2 Fig APE1 chemical shift perturbations induced by 5-fluoroindole-2-carboxylic acid (2). (a) ^15^N-TROSY spectra for APE1 (0.15 mM) in the absence (black) or presence (red) of **2** (1 mM). (b) Bar chart of chemical shift perturbations (Δδ) for backbone ^1^H, ^15^N resonances (combined) versus amino acid residue. Dashed lines are shown at Δδ values of 0.015 and 0.030. Residues exhibiting Δδ ≥0.015 are labeled in both figures.
(TIF)

S3 Fig APE1 chemical shift perturbations induced by 5-nitroindole-2-carboxylic acid (3). (a) ^15^N-TROSY spectra for APE1 (0.15 mM) in the absence (black) or presence (red) of **3** (1 mM). (b) Bar chart of chemical shift perturbations (Δδ) for backbone ^1^H, ^15^N resonances (combined) versus amino acid residue. Dashed lines are shown at Δδ values of 0.015 and 0.030. Residues exhibiting Δδ ≥0.015 are labeled in both figures.
(TIF)

S4 Fig APE1 chemical shift perturbations induced by 6-bromoindole-2-carboxylic acid (4). (a) ^15^N-TROSY spectra for APE1 (0.15 mM) in the absence (black) or presence (red) of **4** (1 mM).(b) Bar chart of chemical shift perturbations (Δδ) for backbone ^1^H, ^15^N resonances (combined) versus amino acid residue. Dashed lines are shown at Δδ values of 0.015 and 0.030. Residues exhibiting Δδ ≥0.015 are labeled in both figures.
(TIF)

S5 Fig APE1 chemical shift perturbations induced by 1% DMSO. (a) Bar chart of CSPs (Δδ) versus amino acid residue of APE1. Residues that exhibit Δδ >0.015 ppm are labeled; none exhibit Δδ >0.017 ppm. The data were obtained from ^15^N-TROSY spectra for APE1 (0.10 mM) in the absence or presence of 1% DMSO. (b) Three residues for which DMSO induces CSPs (Δδ >0.015 ppm) are indicated by blue spheres (backbone N) on a structure of apo APE1-C138A that was determined using crystals that had been soaked in a solution containing 5% DMSO (PDB ID: 6MK3). The two DMSO molecules in this structure are shown in ball and stick format. Residue 140 is near the remote binding pocket identified in this work; residues 69 and 97 are near the DNA binding groove.
(TIF)

S6 Fig New structure of apo human APE1 with a focus on the remote binding pocket. (a) apo human APE1 is shown in cartoon with some side chains and main chain atoms in stick format and select water molecules as red spheres (PDB ID: 7TC3, S1 Table). Dashed lines represent hydrogen bonds with distances shown (Å). The 2*F*_o_-*F*_c_ electron density map, contoured at 1.0 *σ*, is shown for side chains, some mainchain atoms and water molecules. For this model the resolution cutoff was 1.25 Å. (b) The same view of a model that was refined using the same diffraction data but with a resolution cutoff of 1.40 Å. The 2*F*_o_-*F*_c_ electron density map, contoured at 1.0 *σ*, is shown for the same side chains, mainchain atoms, and water molecules as in panel a. The figure shows no significant change in electron density relative to that observed for the model refined with a resolution cutoff of 1.25 Å (panel a). (c) Superposition of our structure of apo APE1 and a prior structure of apo APE1-C138A, which is shown in white with water molecules as magenta spheres (PDB ID: 4QHD). The hydrogen bonds shown are those observed in panels a and b (new structure of apo APE1).
(TIF)

S7 Fig Alignment of structures for apo APE1 and apo APE1 with 5-nitroindole-2-carboxylate. The orientation and coloring are similar to that of Fig 3A in the main text, with APE1 and 5-nitroindole-2-carboxylate in white and cyan, respectively, and water molecules as red spheres for the enzyme-compound complex, and compound-free apo APE1 shown in tan with water molecules as red stars.
(TIF)

S8 Fig APE1 chemical shift perturbations induced by compound 6 (MLS000552981). (a) ^15^N-TROSY spectra for APE1 (0.10 mM) in the absence (black) or presence (red) of compound **6** (0.30 mM). (b) Bar chart of chemical shift perturbations (Δδ) for backbone ^1^H, ^15^N resonances (combined) versus amino acid residue.
(TIF)

S9 Fig APE1 chemical shift perturbations induced by compound 7 (MLS000419194). (a) ^15^N-TROSY spectra for APE1 (0.05 mM) in the absence (black) or presence (red) of **7** (0.03 mM). (b) Bar chart of chemical shift perturbations (Δδ) for backbone ^1^H, ^15^N resonances (combined) versus amino acid residue. Residues exhibiting Δδ ≥ 0.015 ppm are labeled. Both NMR samples contained 0.05% Brij 35, which does alter the spectra of APE1 in the absence of ligand but reduces aggregation of compound **7**.
(TIF)

S10 Fig APE1 chemical shift perturbations induced by MgCl_2_ (0.25 mM). (a) ^15^N-TROSY spectra for APE1 (0.10 mM) in the absence (black) or presence (red) of MgCl_2_ (0.25 mM). Spectra were also collected for APE1 with [MgCl_2_] at 0.063 and 0.125 mM. Two residues near the Mg^2+^-binding site (69, 308) exhibit peaks for apo APE1 but not APE1 with MgCl_2_ (≥0.063 mM). (b) CSPs (Δδ) induced by MgCl_2_ (0.25 mM) as a function of amino acid residue. Labels with one or more stars denote residues for which a peak is not seen in spectra collected for APE1 with 0.25 mM MgCl_2_; for these residues, Δδ values were calculated using spectra for APE1 with the highest [MgCl_2_] for which the peak is observed (*, 0.063 mM; **, 0.125 mM). Residues exhibiting Δδ ≥ 0.015 ppm are labeled in both figures.
(TIF)

S11 Fig APE1 chemical shift perturbations induced by MgCl_2_ (1.0 mM). (a) ^15^N-TROSY spectra for APE1 (0.10 mM) in the absence (black) or presence (red) of MgCl_2_ (1.0 mM). Spectra were also collected for APE1 with lower MgCl_2_ concentrations (0.063, 0.125, 0.25, 0.50. 0.75 mM). Two residues near the Mg^2+^-binding site (69, 308) exhibit peaks for apo APE1 but not APE1 with MgCl_2_ (≥0.063 mM). (b) CSPs (Δδ) induced by MgCl_2_ (1.0 mM) versus amino acid residue. Labels with stars mark residues for which a peak is not seen in spectra of APE1 with 1.0 mM MgCl_2_; for these residues, Δδ values were calculated using spectra for APE1 with the highest [MgCl_2_] for which that peak is observed (*, 0.063 mM; **, 0.125 mM; ***, 0.25 mM; ****, 0.50 mM; *****, 0.75 mM). Residues exhibiting Δδ ≥ 0.015 ppm are labeled.
(TIF)

S12 Fig NMR perturbations for Mg^2+^-APE1 as induced by compound 6 (MLS000552981). NMR experiments were performed using 0.30 mM compound **6** and 0.10 mM APE1 in the presence of MgCl_2_ at a concentration of (a) 1.0 mM or (b) 0.25 mM.
(TIF)

S13 Fig NMR perturbations for Mg^2+^-APE1 as induced by compound 7 (MLS000419194). NMR experiments were performed using 0.030 mM compound **7** and 0.05 mM APE1 in the presence of MgCl_2_ at a concentration of (a) 1.0 mM or (b) 0.25 mM. The NMR samples also contained detergent (0.05% Brij 35) which helps to preclude aggregation of **7** but does alter the spectra of APE1 in the absence of ligand.
(TIF)

S1 Table Data collection and refinement statistics. Values shown in parenthesis are for highest resolution shell. The Ramachandran analysis was performed using Procheck [74].
(DOCX)

S1 File (MTZ)

S2 File (PDB)

S3 File (MTZ)

S4 File (PDB)
