# Supplementary figures and images for: Characterizing inhibitors of human AP endonuclease 1
Source: PLoS One. 2023 Jan 18;18(1):e0280526. doi: 10.1371/journal.pone.0280526 (PMC9847973; doi:10.1371/journal.pone.0280526)

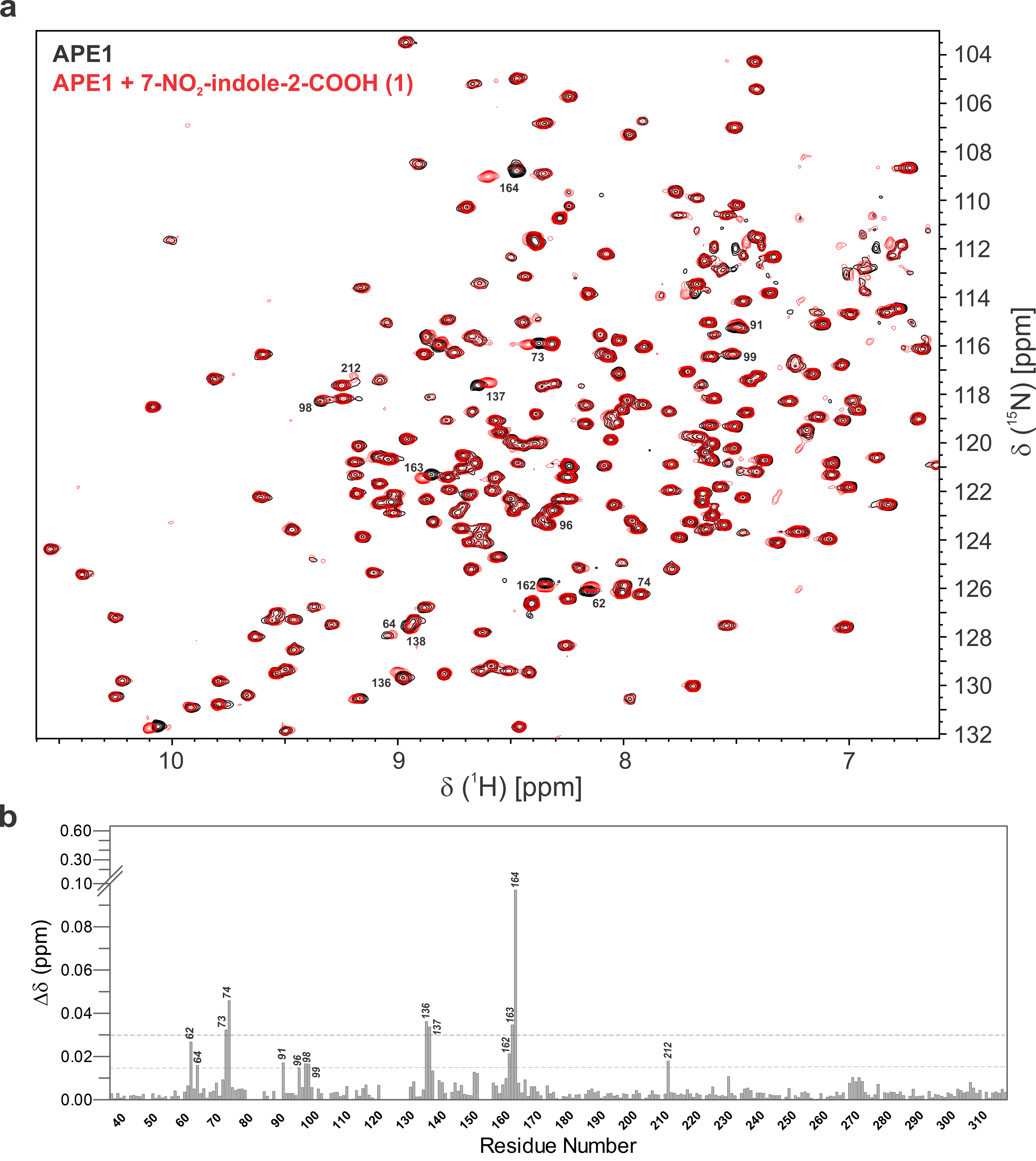

Supplement: S1 Fig — (a) 15N-TROSY spectra for APE1 (0.15 mM) in the absence (black) or presence (red) of 1 (1 mM). (b) Bar chart of chemical shift perturbations (Δδ) for backbone 1H, 15N resonances (combined) versus amino acid residue. Dashed lines are shown at Δδ values of 0.015 and 0.030. Residues exhibiting Δδ ≥0.015 are labeled in both figures. (TIF) [file pone.0280526.s001.tif]

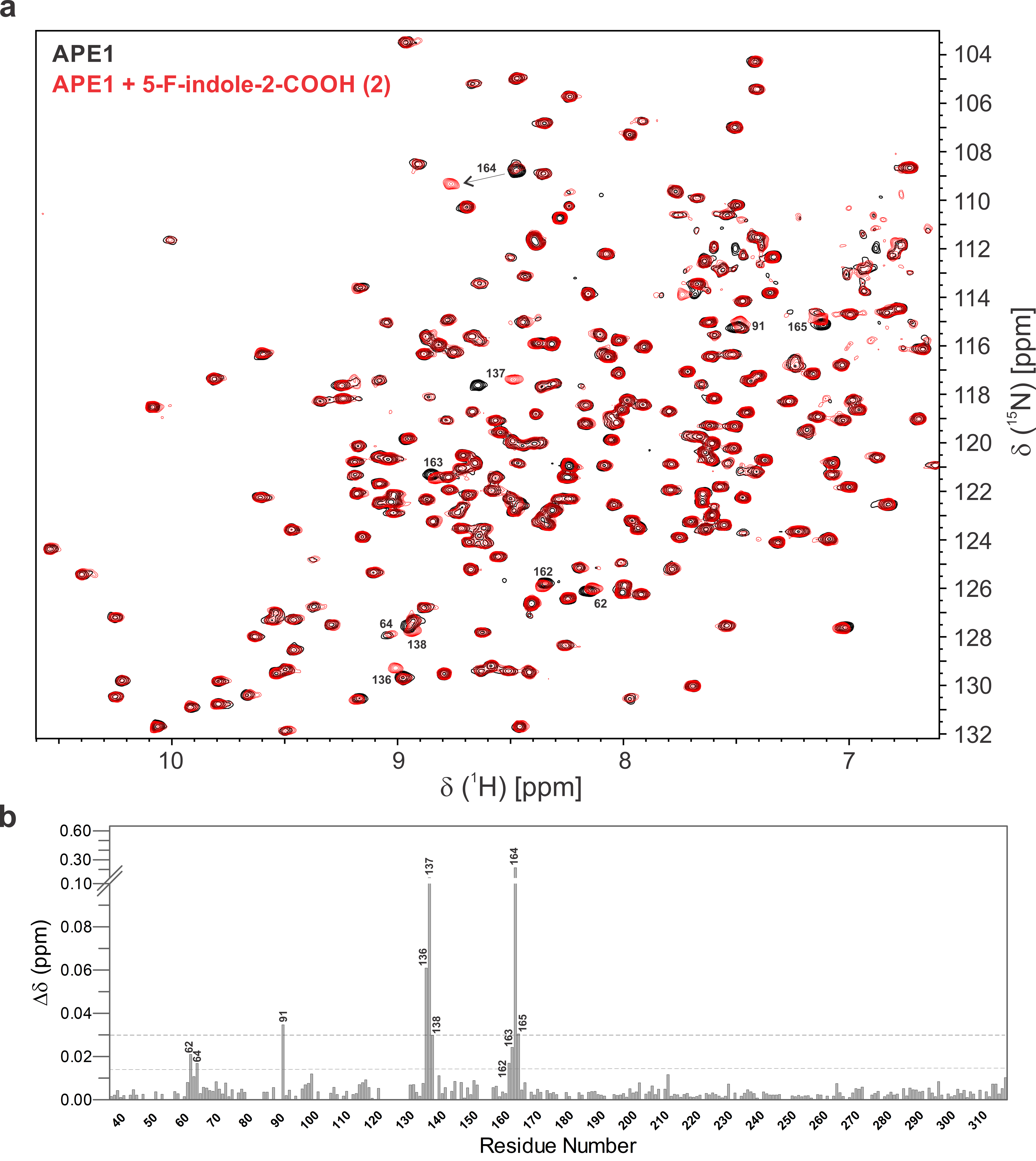

Supplement: S2 Fig — (a) 15N-TROSY spectra for APE1 (0.15 mM) in the absence (black) or presence (red) of 2 (1 mM). (b) Bar chart of chemical shift perturbations (Δδ) for backbone 1H, 15N resonances (combined) versus amino acid residue. Dashed lines are shown at Δδ values of 0.015 and 0.030. Residues exhibiting Δδ ≥0.015 are labeled in both figures. (TIF) [file pone.0280526.s002.tif]

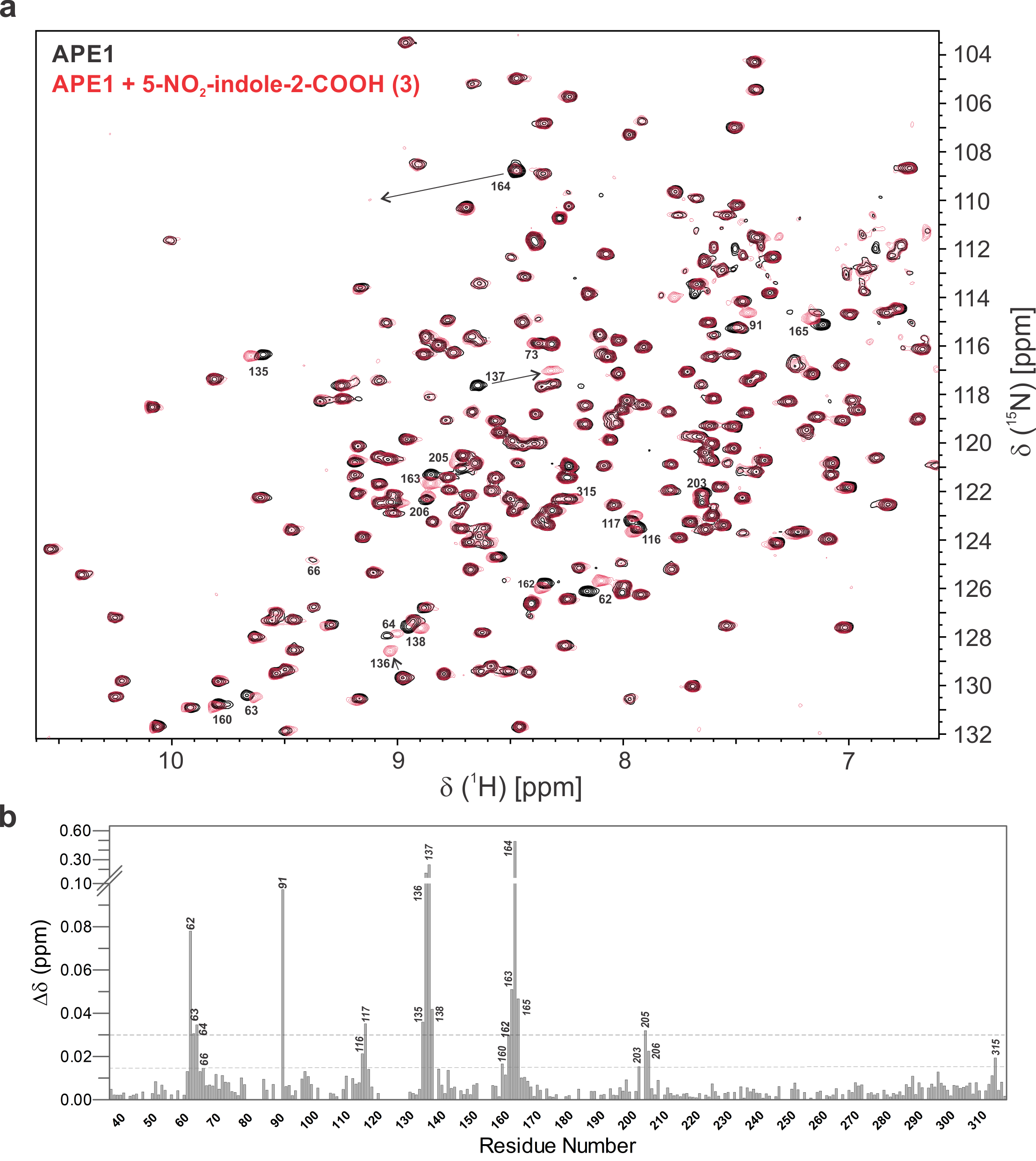

Supplement: S3 Fig — (a) 15N-TROSY spectra for APE1 (0.15 mM) in the absence (black) or presence (red) of 3 (1 mM). (b) Bar chart of chemical shift perturbations (Δδ) for backbone 1H, 15N resonances (combined) versus amino acid residue. Dashed lines are shown at Δδ values of 0.015 and 0.030. Residues exhibiting Δδ ≥0.015 are labeled in both figures. (TIF) [file pone.0280526.s003.tif]

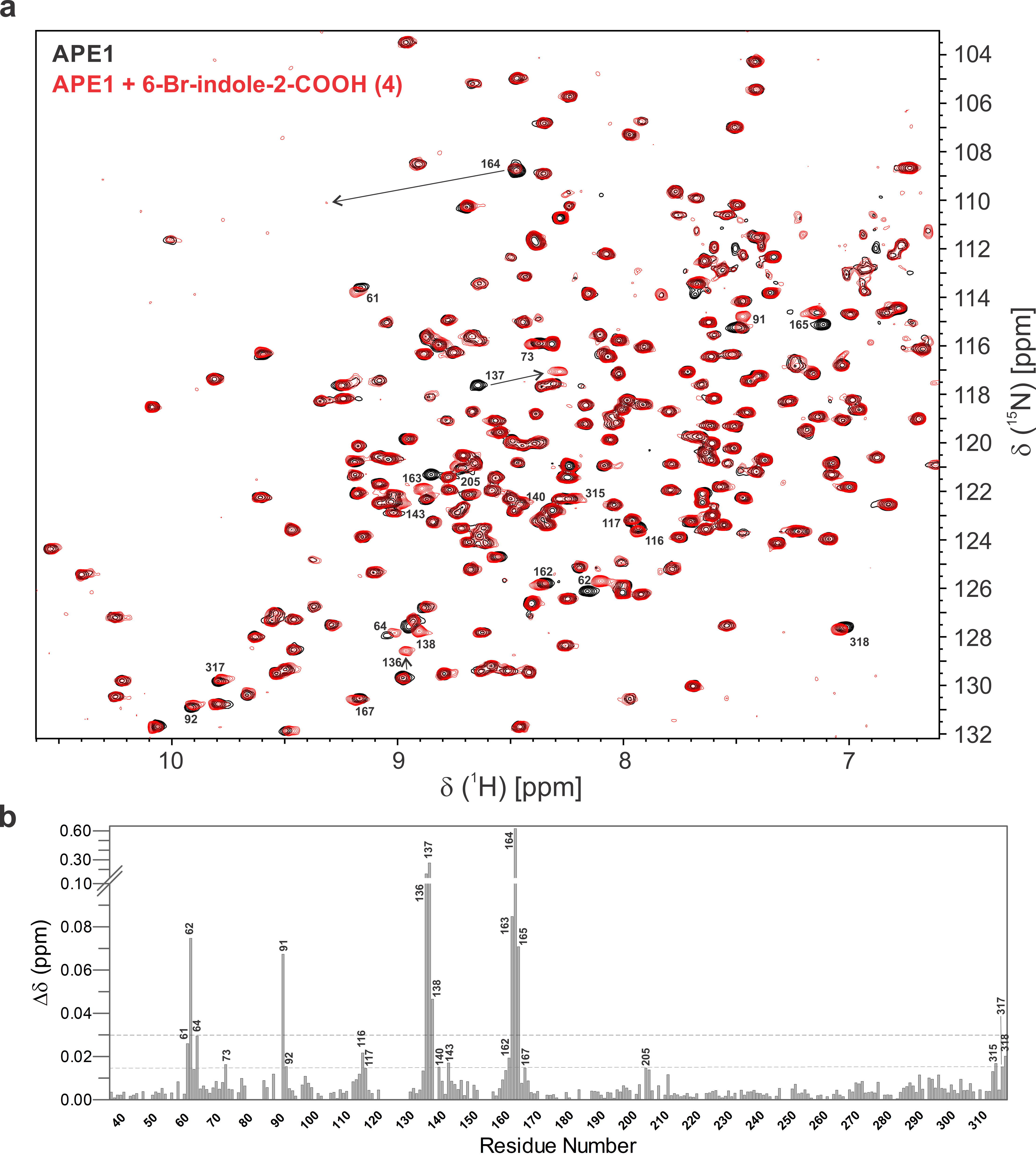

Supplement: S4 Fig — (a) 15N-TROSY spectra for APE1 (0.15 mM) in the absence (black) or presence (red) of 4 (1 mM).(b) Bar chart of chemical shift perturbations (Δδ) for backbone 1H, 15N resonances (combined) versus amino acid residue. Dashed lines are shown at Δδ values of 0.015 and 0.030. Residues exhibiting Δδ ≥0.015 are labeled in both figures. (TIF) [file pone.0280526.s004.tif]

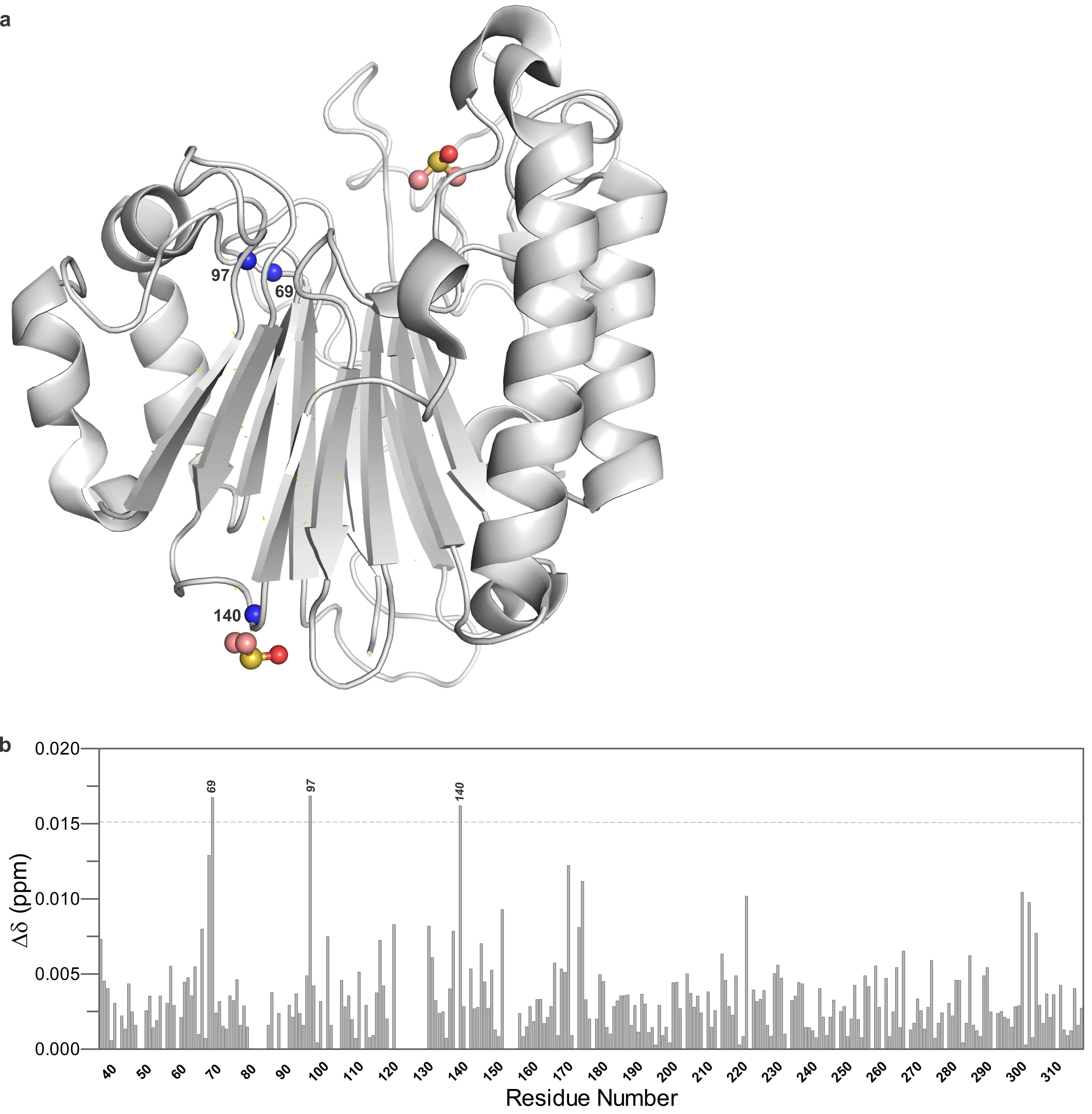

Supplement: S5 Fig — (a) Bar chart of CSPs (Δδ) versus amino acid residue of APE1. Residues that exhibit Δδ >0.015 ppm are labeled; none exhibit Δδ >0.017 ppm. The data were obtained from 15N-TROSY spectra for APE1 (0.10 mM) in the absence or presence of 1% DMSO. (b) Three residues for which DMSO induces CSPs (Δδ >0.015 ppm) are indicated by blue spheres (backbone N) on a structure of apo APE1-C138A that was determined using crystals that had been soaked in a solution containing 5% DMSO (PDB ID: 6MK3). The two DMSO molecules in this structure are shown in ball and stick format. Residue 140 is near the remote binding pocket identified in this work; residues 69 and 97 are near the DNA binding groove. (TIF) [file pone.0280526.s005.tif]

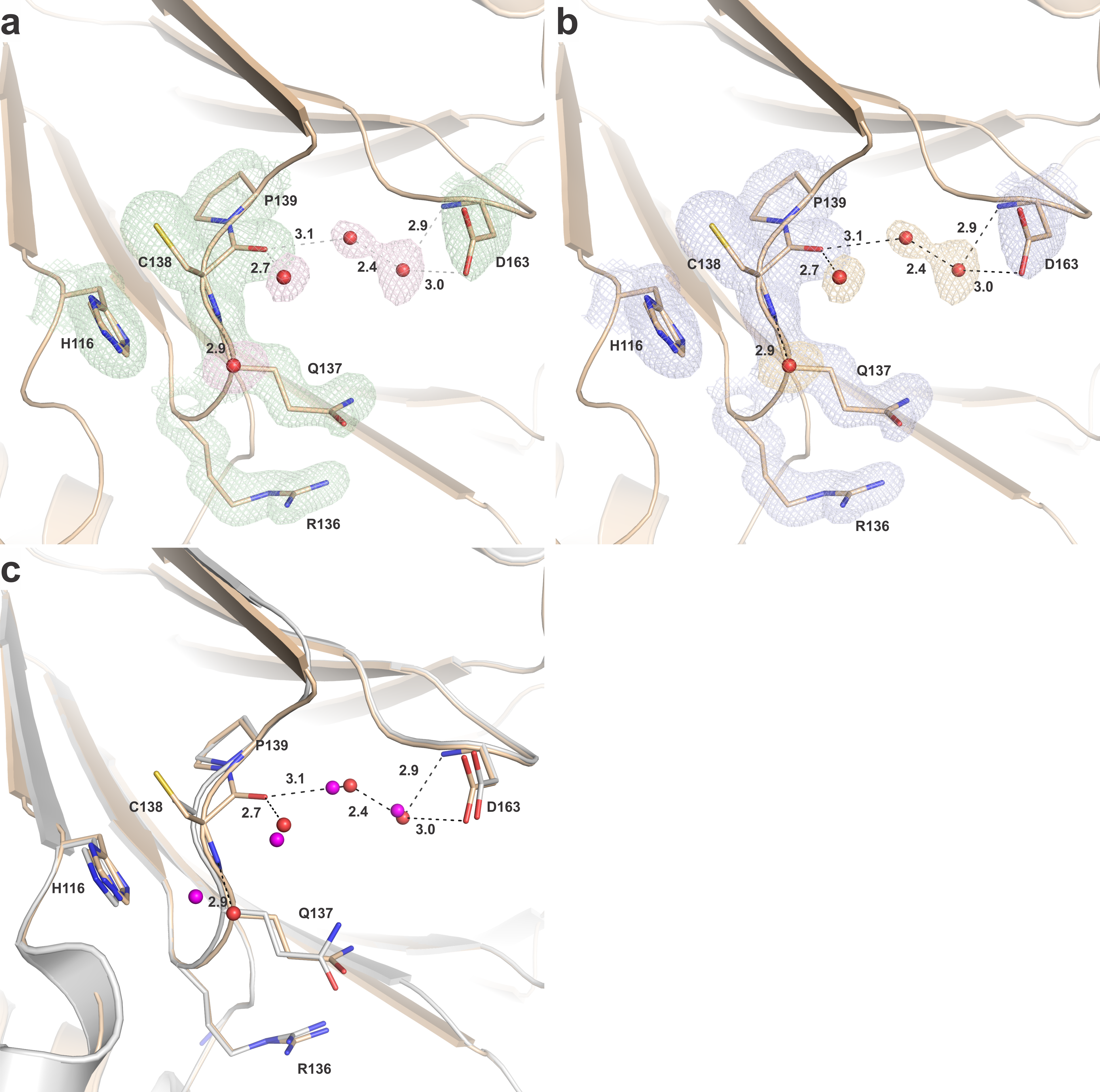

Supplement: S6 Fig — (a) apo human APE1 is shown in cartoon with some side chains and main chain atoms in stick format and select water molecules as red spheres (PDB ID: 7TC3, S1 Table). Dashed lines represent hydrogen bonds with distances shown (Å). The 2Fo-Fc electron density map, contoured at 1.0 σ, is shown for side chains, some mainchain atoms and water molecules. For this model the resolution cutoff was 1.25 Å. (b) The same view of a model that was refined using the same diffraction data but with a resolution cutoff of 1.40 Å. The 2Fo-Fc electron density map, contoured at 1.0 σ, is shown for the same side chains, mainchain atoms, and water molecules as in panel a. The figure shows no significant change in electron density relative to that observed for the model refined with a resolution cutoff of 1.25 Å (panel a). (c) Superposition of our structure of apo APE1 and a prior structure of apo APE1-C138A, which is shown in white with water molecules as magenta spheres (PDB ID: 4QHD). The hydrogen bonds shown are those observed in panels a and b (new structure of apo APE1). (TIF) [file pone.0280526.s006.tif]

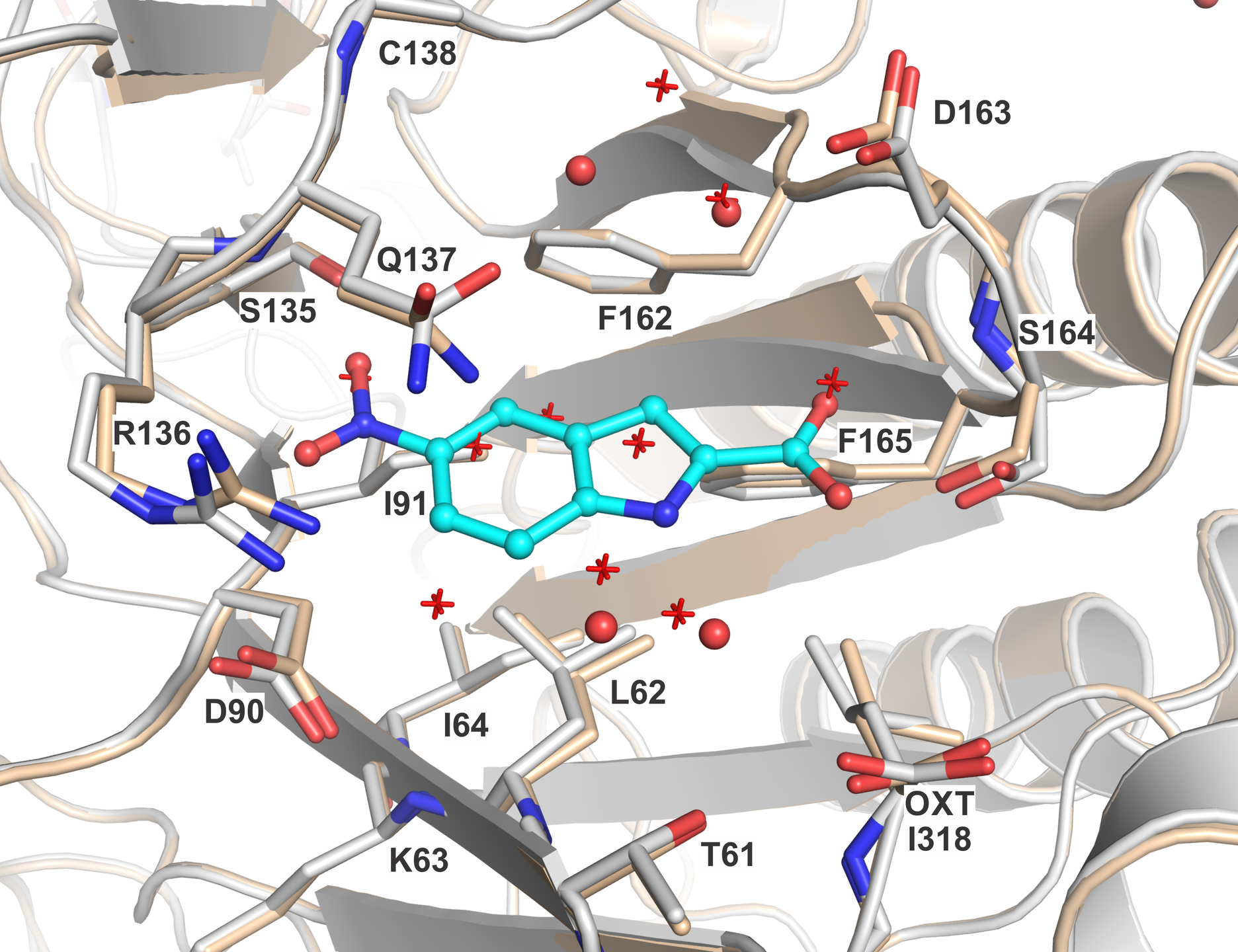

Supplement: S7 Fig — The orientation and coloring are similar to that of Fig 3A in the main text, with APE1 and 5-nitroindole-2-carboxylate in white and cyan, respectively, and water molecules as red spheres for the enzyme-compound complex, and compound-free apo APE1 shown in tan with water molecules as red stars. (TIF) [file pone.0280526.s007.tif]

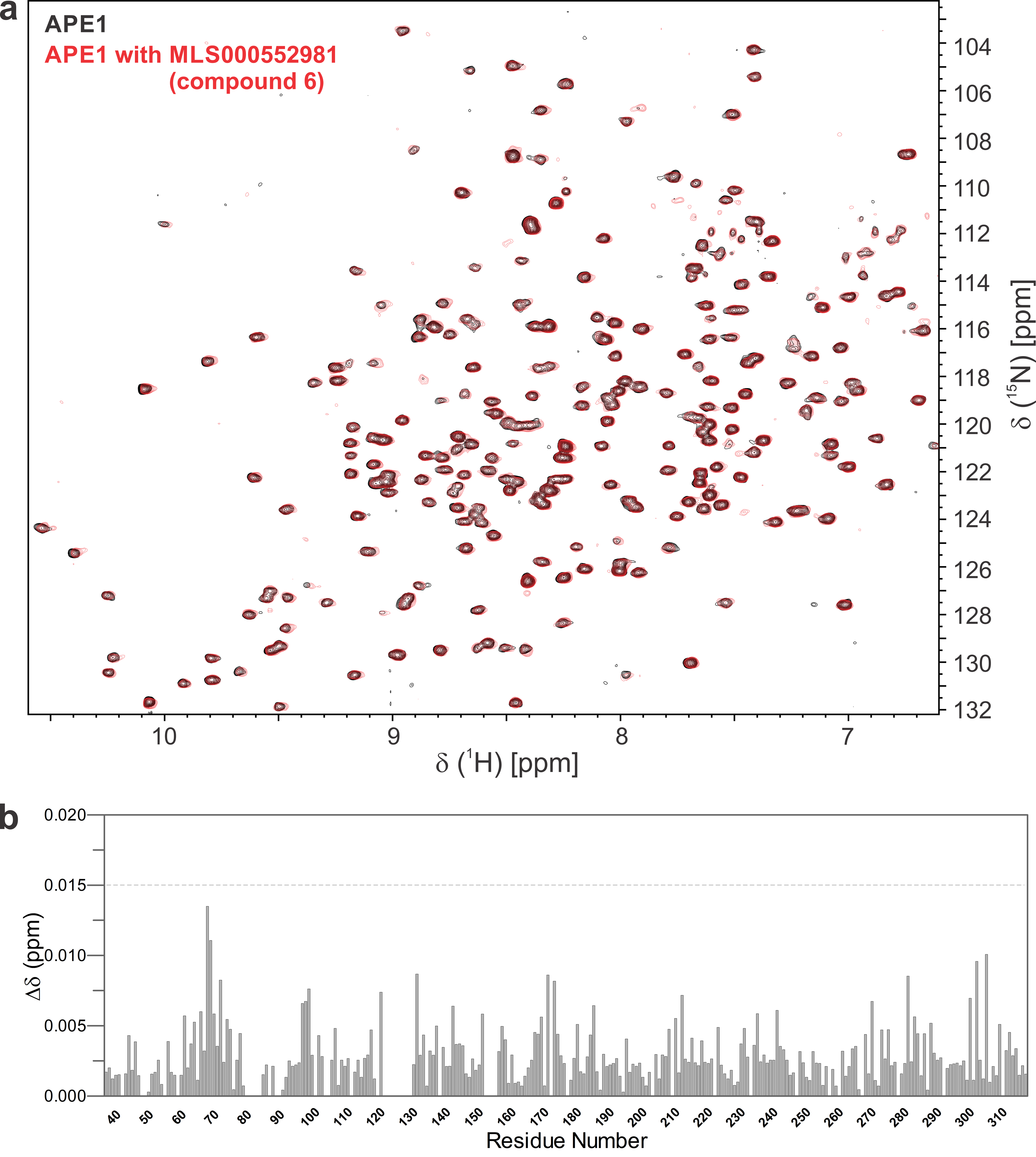

Supplement: S8 Fig — (a) 15N-TROSY spectra for APE1 (0.10 mM) in the absence (black) or presence (red) of compound 6 (0.30 mM). (b) Bar chart of chemical shift perturbations (Δδ) for backbone 1H, 15N resonances (combined) versus amino acid residue. (TIF) [file pone.0280526.s008.tif]

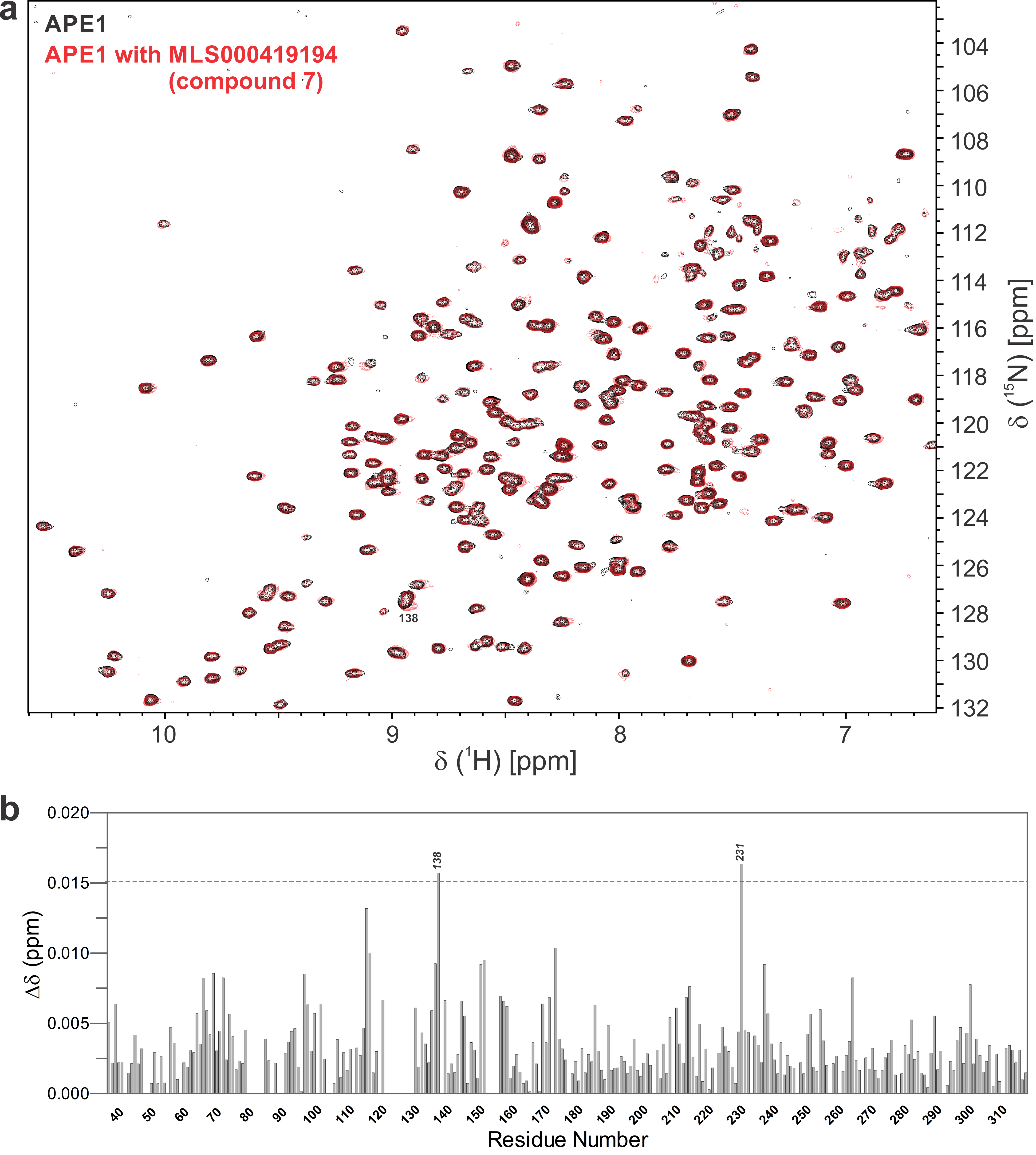

Supplement: S9 Fig — (a) 15N-TROSY spectra for APE1 (0.05 mM) in the absence (black) or presence (red) of 7 (0.03 mM). (b) Bar chart of chemical shift perturbations (Δδ) for backbone 1H, 15N resonances (combined) versus amino acid residue. Residues exhibiting Δδ ≥ 0.015 ppm are labeled. Both NMR samples contained 0.05% Brij 35, which does alter the spectra of APE1 in the absence of ligand but reduces aggregation of compound 7. (TIF) [file pone.0280526.s009.tif]

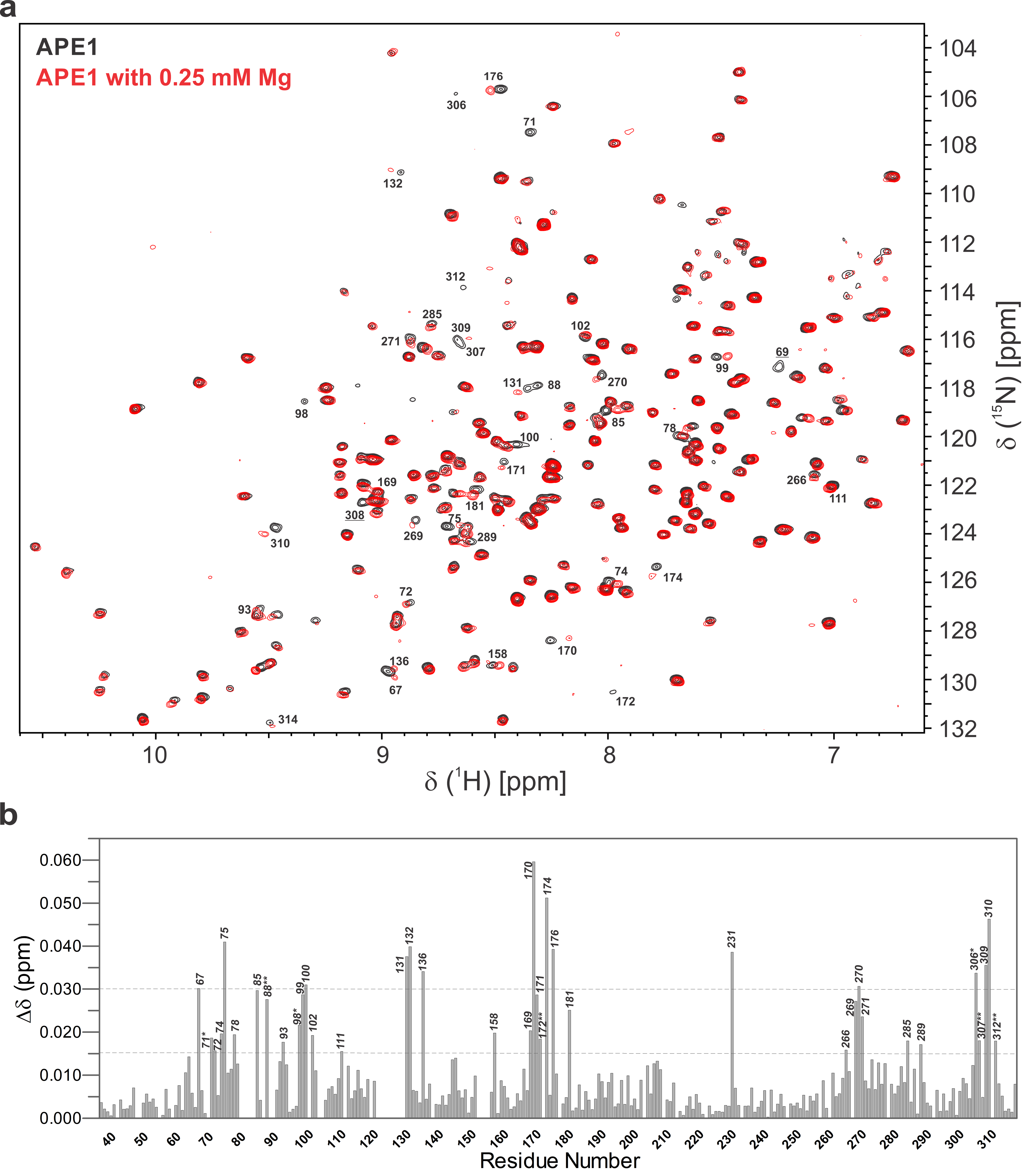

Supplement: S10 Fig — (a) 15N-TROSY spectra for APE1 (0.10 mM) in the absence (black) or presence (red) of MgCl2 (0.25 mM). Spectra were also collected for APE1 with [MgCl2] at 0.063 and 0.125 mM. Two residues near the Mg2+-binding site (69, 308) exhibit peaks for apo APE1 but not APE1 with MgCl2 (≥0.063 mM). (b) CSPs (Δδ) induced by MgCl2 (0.25 mM) as a function of amino acid residue. Labels with one or more stars denote residues for which a peak is not seen in spectra collected for APE1 with 0.25 mM MgCl2; for these residues, Δδ values were calculated using spectra for APE1 with the highest [MgCl2] for which the peak is observed (*, 0.063 mM; **, 0.125 mM). Residues exhibiting Δδ ≥ 0.015 ppm are labeled in both figures. (TIF) [file pone.0280526.s010.tif]

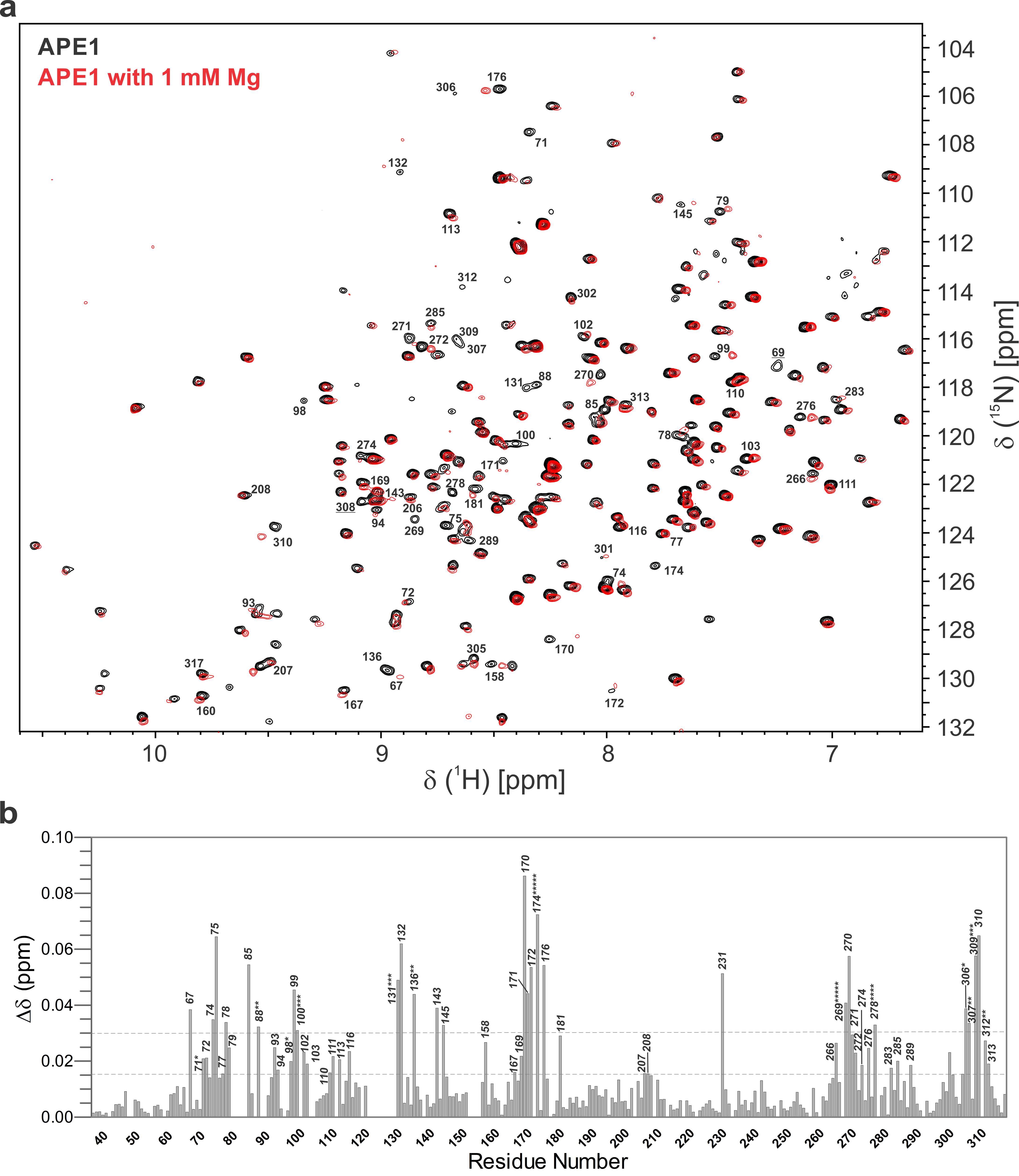

Supplement: S11 Fig — (a) 15N-TROSY spectra for APE1 (0.10 mM) in the absence (black) or presence (red) of MgCl2 (1.0 mM). Spectra were also collected for APE1 with lower MgCl2 concentrations (0.063, 0.125, 0.25, 0.50. 0.75 mM). Two residues near the Mg2+-binding site (69, 308) exhibit peaks for apo APE1 but not APE1 with MgCl2 (≥0.063 mM). (b) CSPs (Δδ) induced by MgCl2 (1.0 mM) versus amino acid residue. Labels with stars mark residues for which a peak is not seen in spectra of APE1 with 1.0 mM MgCl2; for these residues, Δδ values were calculated using spectra for APE1 with the highest [MgCl2] for which that peak is observed (*, 0.063 mM; **, 0.125 mM; ***, 0.25 mM; ****, 0.50 mM; *****, 0.75 mM). Residues exhibiting Δδ ≥ 0.015 ppm are labeled. (TIF) [file pone.0280526.s011.tif]

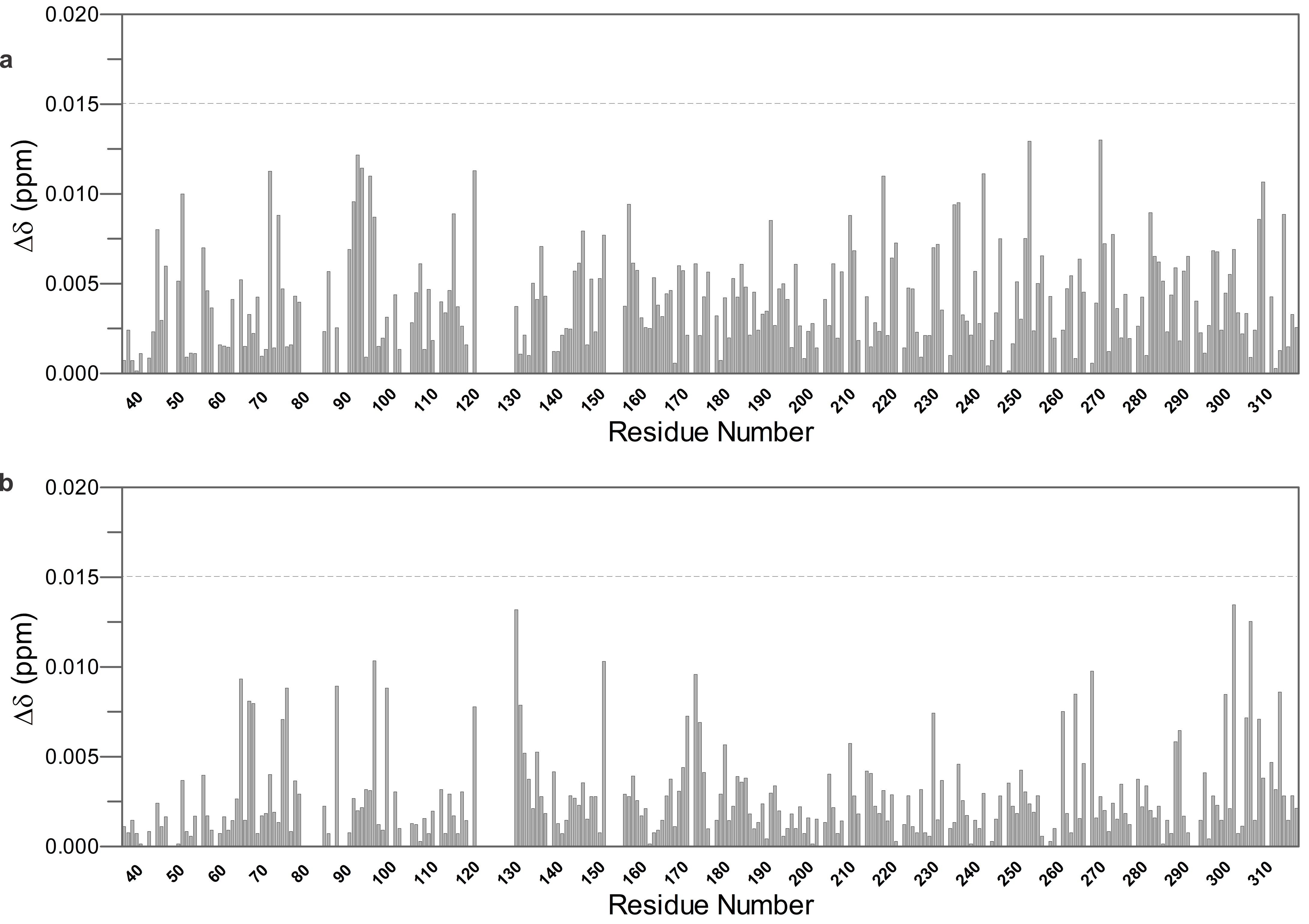

Supplement: S12 Fig — NMR experiments were performed using 0.30 mM compound 6 and 0.10 mM APE1 in the presence of MgCl2 at a concentration of (a) 1.0 mM or (b) 0.25 mM. (TIF) [file pone.0280526.s012.tif]

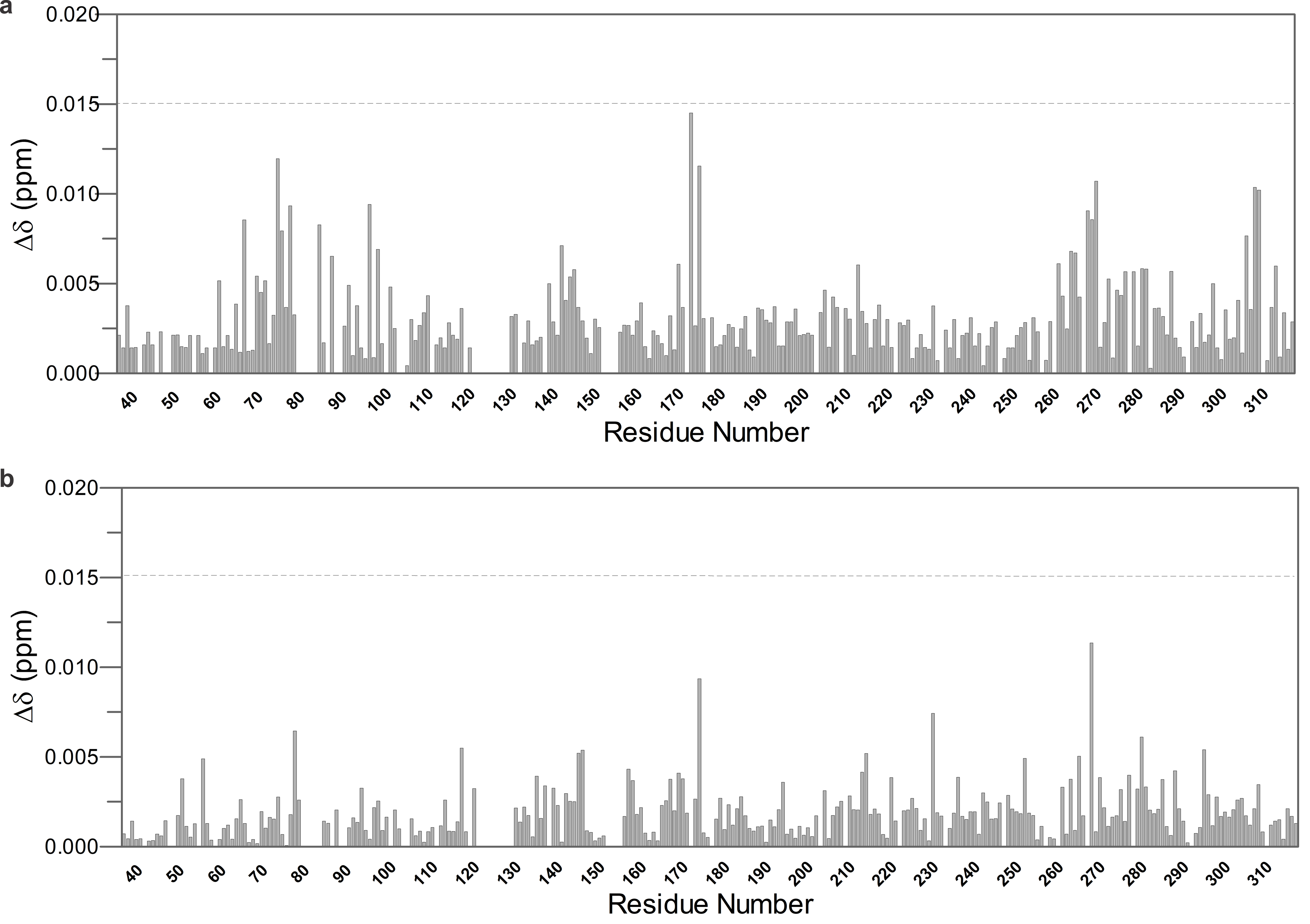

Supplement: S13 Fig — NMR experiments were performed using 0.030 mM compound 7 and 0.05 mM APE1 in the presence of MgCl2 at a concentration of (a) 1.0 mM or (b) 0.25 mM. The NMR samples also contained detergent (0.05% Brij 35) which helps to preclude aggregation of 7 but does alter the spectra of APE1 in the absence of ligand. (TIF) [file pone.0280526.s013.tif]
