# Supplementary material for: Characterizing inhibitors of human AP endonuclease 1
Source: PLoS One. 2023 Jan 18;18(1):e0280526. doi: 10.1371/journal.pone.0280526 (PMC9847973; doi:10.1371/journal.pone.0280526)
Supplement: S1 Table — Values shown in parenthesis are for highest resolution shell. The Ramachandran analysis was performed using Procheck [74]. (DOCX) [file pone.0280526.s014.docx]

|  | | APE1 + 5-NO_2_-indole-2-COOH | apo APE1 |
| --- | --- | --- | --- |
|  | (PDB ID: 7tc2) | | (PDB ID: 7tc3) |
| **Data collection** |  | |  |
| Space Group | P2_1_ | | P2_1_ 2_1_ 2 |
| Cell Dimensions |  | |  |
| *a, b, c* (Å), β(°) | 46.65, 140.78, 89.57, 92.9 | | 46.65, 141.42, 45.26, 90.0 |
| Resolution (Å) | 38.90-1.43 (1.46-1.43) | | 38.94-1.25 (1.27-1.25) |
| *R*_pim_ | | 0.050 | 0.013 |
| Mean *I*/σ*I* | | 6.8 (0.6) | 22.6 (0.5) |
| CC_1/2_ | | 0.996 (0.290) | 1.000 (0.166) |
| Completeness (%) | | 98.6 (98.2) | 98.9 (78.9) |
| Redundancy | | 6.8 (6.4) | 10.8 (1.7) |
| Wilson B-factor (Å^2^) | | 21.1 | 15.5 |
|  | |  |  |
| **Refinement** | |  |  |
| Program | | Buster | Phenix |
| Resolution (Å) | | 38.90-1.43 | 38.94-1.25 |
| No. of reflections | | 206075 | 82165 |
| *R*_work_/*R*_free_ | | 0.251/0.270 | 0.151/0.178 |
| Number of atoms | |  |  |
| protein | | 8664 | 2258 |
| water | | 832 | 398 |
| other | | 75 | 24 |
| B-factors (Å^2^) | |  |  |
| protein | | 27.6 | 27.0 |
| water | | 35.6 | 49.4 |
| ions | | 37.8 | - |
| Ramachandran Plot | |  |  |
| favoured (%) | | 96.9 | 99.3 |
| allowed (%) | | 3.1 | 0.7 |
| outliers (%) | | 0 | 0.0 |
| RMSD from ideal | |  |  |
| bond lengths (Å) | | 0.009 | 0.005 |
| bond angles (°) | | 0.99 | 0.84 |
|  | |  |  |
|  | |  |  |
